# The Role of *Treponema denticola* Motility in Synergistic Biofilm Formation With *Porphyromonas gingivalis*

**DOI:** 10.3389/fcimb.2019.00432

**Published:** 2019-12-18

**Authors:** Hong Min Ng, Nada Slakeski, Catherine A. Butler, Paul D. Veith, Yu-Yen Chen, Sze Wei Liu, Brigitte Hoffmann, Stuart G. Dashper, Eric C. Reynolds

**Affiliations:** Oral Health Cooperative Research Centre, Melbourne Dental School, Bio21 Institute, The University of Melbourne, Melbourne, VIC, Australia

**Keywords:** polymicrobial biofilm, chronic periodontitis, periplasmic flagella, motility, quantitative proteomics

## Abstract

Chronic periodontitis has a polymicrobial biofilm etiology and interactions between key oral bacterial species, such as *Porphyromonas gingivalis* and *Treponema denticola* contribute to disease progression. *P. gingivalis* and *T. denticola* are co-localized in subgingival plaque and have been previously shown to exhibit strong synergy in growth, biofilm formation and virulence in an animal model of disease. The motility of *T. denticola*, although not considered as a classic virulence factor, may be involved in synergistic biofilm development between *P. gingivalis* and *T. denticola*. We determined the role of *T. denticola* motility in polymicrobial biofilm development using an optimized transformation protocol to produce two *T. denticola* mutants targeting the motility machinery. These deletion mutants were non-motile and lacked the gene encoding the flagellar hook protein of the periplasmic flagella (Δ*flgE*) or a component of the stator motor that drives the flagella (Δ*motB*). The specificity of these gene deletions was determined by whole genome sequencing. Quantitative proteomic analyses of mutant strains revealed that the specific inactivation of the motility-associated gene, *motB*, had effects beyond motility. There were 64 and 326 proteins that changed in abundance in the Δ*flgE* and Δ*motB* mutants, respectively. In the Δ*flgE* mutant, motility-associated proteins showed the most significant change in abundance confirming the phenotype change for the mutant was related to motility. However, the inactivation of *motB* as well as stopping motility also upregulated cellular stress responses in the mutant indicating pleiotropic effects of the mutation. *T. denticola* wild-type and *P. gingivalis* displayed synergistic biofilm development with a 2-fold higher biomass of the dual-species biofilms than the sum of the monospecies biofilms. Inactivation of *T. denticola flgE* and *motB* reduced this synergy. A 5-fold reduction in dual-species biofilm biomass was found with the motility-specific Δ*flgE* mutant suggesting that *T. denticola* periplasmic flagella are essential in synergistic biofilm formation with *P. gingivalis*.

## Introduction

Chronic periodontitis is a polymicrobial disease believed to be initiated by changes in the bacterial species composition of subgingival plaque biofilms, and subsequent dysregulation of the host immune response (Hajishengallis and Lamont, 2012). It is linked with the overgrowth of a small number of oral microbial species within subgingival plaque biofilms accreted to the surface of the tooth root (Wiebe and Putnins, 2000; Byrne et al., 2009). *Treponema denticola* and *Porphyromonas gingivalis* are pathobionts associated with chronic periodontitis due to their strong association with the clinical measurements of severe periodontal disease, such as periodontal pocket depth and bleeding on probing (Lamont and Jenkinson, 1998; Socransky et al., 1998; Holt and Ebersole, 2005; Dashper et al., 2011). The study of their interactions in polymicrobial biofilms is important to understand chronic disease initiation and progression.

*T. denticola* and *P. gingivalis* synergistically form biofilms *in vitro* (Yamada et al., 2005; Zhu et al., 2013). Intriguingly, *T. denticola* cells in monospecies biofilms lose their characteristic spiral morphology but retain it when grown in polymicrobial biofilms with *P. gingivalis* (Zhu et al., 2013). The spiral morphology of *T. denticola* is closely related to its periplasmic flagella (Ruby et al., 1997), which in turn are required for motility (Li et al., 1996). *T. denticola* motility therefore may play a role in synergistic biofilm formation with *P. gingivalis*. Unlike extracellularly flagellated bacteria, *T. denticola* remains motile in a highly viscous environment, likely due to the protection of the flagella in the periplasmic space, away from direct contact with the external environment (Klitorinos et al., 1993). The ability of *T. denticola* to move in highly viscous environments may be beneficial for its movement through polymicrobial biofilms. Together with the presence of a chemotaxis system that allows it to move in response to environmental stimuli, *T. denticola* may create pores in the biofilm matrix as they move through the biofilms (Houry et al., 2012), allowing better nutrient penetration and waste removal, thereby contributing to a larger biofilm biomass.

**Figure 1:**
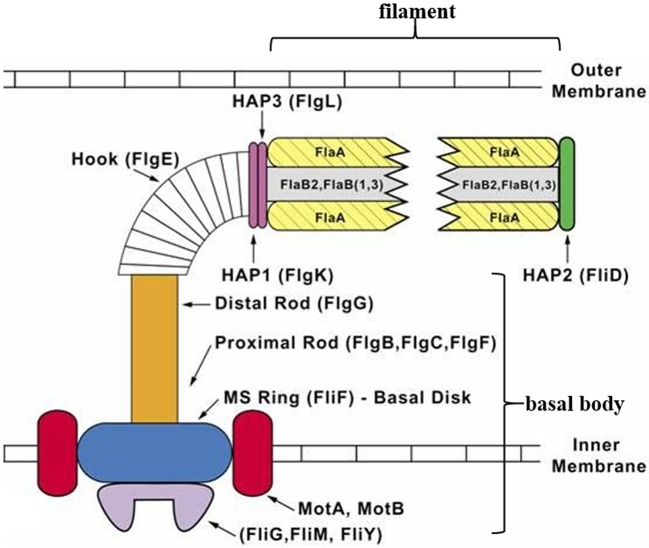
Diagrammatic representation of a *T. denticola* periplasmic flagellum. The periplasmic flagellum is made up of three parts: a basal body, hook, and filament. The flagellar hook (FlgE) connects and transmits torque produced by the motor [rotor (FliF, FliG, FliM, and FliY) and stator (MotA and MotB)] in the basal body, to the flagellar filament which then rotates. Diagram was adapted and modified from Limberger (2004).

Similar to a typical bacterial flagellum, a spirochete periplasmic flagellum can be divided into three parts: a basal body, hook, and filament (Figure 1). The basal body is composed of a rod and a motor-switch complex embedded in the inner membrane. The motor is made up of two parts: the rotor and the stator. The rotor is made up of proteins FliF, FliG, FliM, and FliY (Limberger, 2004; Morimoto and Minamino, 2014). The stator, which is comprised of two integral membrane proteins (MotA and MotB), is an ion channel complex coupling transmembrane ion movement with flagellar rotation (Kojima and Blair, 2001; Chevance and Hughes, 2008; Kojima et al., 2009; Morimoto and Minamino, 2014). The flagellar hook structure consists of one major polypeptide, FlgE, and serves as a universal joint that transmits torque produced by the motor in the basal body to the flagellar filament, whose rotation results in specific movements of the cell body that enables the cell to move (Berg, 1976; Limberger, 2004). The flagellar filament of *T. denticola* is made up of three filament outer layer proteins (FlaA1-3) and three filament core proteins (FlaB1-3). The flagellar filaments originate at opposite ends of the same cell and overlap at mid-cell (Chan et al., 1993). Each *T. denticola* cell commonly possesses four periplasmic flagella, two anchored at each end of the cell that overlap in the middle of the cell (Izard et al., 2008).

*T. denticola* is a slow-growing, fastidious and highly fragile obligate anaerobe (Salvador et al., 1987; Wardle, 1997), making its cultivation and handling in the laboratory difficult. The characterization of the molecular detail of *T. denticola* virulence has been hampered by its low transformation efficiency, lack of shuttle plasmids and small number of selectable markers (Li and Kuramitsu, 1996; Li et al., 1996, 2015; Chi et al., 1999; Kuramitsu et al., 2005; Bian and Li, 2011; Godovikova et al., 2015). Despite two decades of work a relatively small number of *T. denticola* transformants have been reported in the literature and many laboratories have reported modifications of the original published protocol for successful transformation (Ishihara et al., 1998; Limberger et al., 1999; Chi et al., 2002; Lux et al., 2002; Abiko et al., 2014; Godovikova et al., 2015).

In this study, we generated two *T. denticola* motility mutants to investigate the role of *T. denticola* motility in synergistic biofilm formation with *P. gingivalis*. The *flgE* and *motB* genes were targeted as their inactivation in *T. denticola* or other bacteria resulted in mutants with impaired motility (Li et al., 1996; Houry et al., 2010; Sultan et al., 2015). The mutants generated were characterized for their morphology, motility, growth, binding with *P. gingivalis* and protein expression profiles. Finally, the ability of the mutants to form dual-species biofilms with *P. gingivalis* was determined.

## Materials and Methods

### Bacterial Strains and Culture Conditions

*P. gingivalis* W50 and *T. denticola* ATCC 33520 and ATCC 35405 were obtained from the culture collection of the Oral Health Cooperative Research Centre, The University of Melbourne. The bacteria were maintained in an anaerobic workstation (MK3; Don Whitley Scientific) at 37°C. Planktonic *P. gingivalis* cultures were routinely grown in Brain Heart Infusion (BHI) medium containing 37 g/L Brain Heart Infusion (Becton, Dickinson and Company), L-cysteine hydrochloride (0.5 mg/mL), and hemin (5 μg/mL)*. T. denticola* was grown in Oral Bacterium Growth Medium (OBGM) (Veith et al., 2009). When needed, agarose (UltraPure™ Low Melting Point Agarose, Thermo Fisher Scientific Inc., USA) and antibiotics (described in detail below) were added to the medium. Growth of bacterial cultures was monitored by measuring absorbance at a wavelength of 650 nm (A_650_) and cells were harvested by centrifugation. Culture purity was routinely checked by Gram staining and colony morphology.

### Construction of *T. denticola* Motility Mutants

*T. denticola* ATCC 33520 motility mutants were constructed where the open reading frame of *flgE* and *motB* were deleted. These two genes are found in the same operon driven by the *fla* promoter; recombination cassettes included the *fla* promoter downstream of the erythromycin resistance gene to enable transcription of the remainder of the operon. Briefly, recombination cassettes for the deletion of *flgE* (*HMPREF9722_RS03025*) and *motB* (*HMPREF9722_RS03040*) were constructed using the PCR-based splicing by overlap-extension (SOE) method (Horton et al., 1989). *T. denticola* genomic DNA was purified using the DNeasy® Blood and Tissue kit (QIAGEN Pty.) according to the manufacturer's instructions. Genomic DNA was resuspended in deionized water and subject to PCR using the primers shown in Supplementary Table 1. A schematic representation of the mutational approach is shown in Supplementary Figure 1. The upstream and downstream regions of the target gene as well as *fla* promoter were amplified by PCR from the chromosomal DNA of *T. denticola* 33520 whilst the *ermAM* gene was amplified from the shuttle vector pHS17 (Fletcher et al., 1995). The four amplicons were fused into a single fragment using the SOE method (Horton et al., 1989). The final fragment was cloned into a pGEM®-T Easy vector, yielding pHN-Δ*flgE* and pHN-Δ*mot*B. The fidelity of each recombination cassette was confirmed by DNA sequencing.

Each plasmid construct (10 μg) was linearized by digestion with NotI then electroporated into *T. denticola* ATCC 33520 cells as previously published (Li et al., 1996) with some modifications. Briefly, 2-days (exponential phase; A_650_ of 0.16–0.28) *T. denticola* ATCC 33520 cultures were decanted into centrifuge tubes in the anaerobic chamber. The cells were harvested by centrifugation (4,000 *g*, 10 min, 4°C) and the subsequent washing and resuspension steps were conducted anaerobically at 4°C. All resuspension steps were performed using 1 mL pipette tips which had ~1 cm cut off to reduce shear forces on cells. The cells were washed twice instead of three times to reduce handling and loss of cells. Sterile 10% (v/v) glycerol used for all wash and resuspension steps was pre-reduced in the anaerobic chamber for at least 16 h before use. Electroporation was carried out as previously described (Li et al., 1996), typically producing a time constant of 4.0–4.7 ms. *T. denticola* cells were then immediately suspended in 1.2 mL of pre-reduced OBGM in the anaerobic chamber and incubated overnight at 37°C. Transformants were selected on OBGM agar plates containing 0.8% (w/v) agarose supplemented with 40 μg/mL erythromycin. The OBGM agar medium was pre-equilibrated to 37°C before plating of the electroporated cells to avoid thermal shock. To screen for the presence of the appropriate homologous recombination event, the resulting transformants were grown in OBGM (2 mL) containing the appropriate antibiotics whereupon a small volume of culture (~25 μL) was subject to PCR with the appropriate oligonucleotide primers (Supplementary Table 1).

### Genomic Sequencing

Genome sequencing was performed using an Ion Torrent Personal Genome Machine (PGM; Thermo Fisher Scientific,) according to the protocols of the manufacturer unless otherwise stated. Briefly, 1 μg of *T. denticola* genomic DNA was fragmented to ~400 bp using a Covaris M220 Focused-ultrasonicator™ (TrendBio, Australia). A 1 μL aliquot of sheared DNA was visualized using a LabChip GX Touch 24 Nucleic Acid Analyzer (PerkinElmer, USA) to ensure a peak fragment size of 400 bp. The DNA was end-repaired (Ion Xpress™ Plus Fragment Library Kit) and purified (Agencourt™ AMPure™ XP Kit, Beckman Coulter). Barcoded adaptors were ligated to the DNA and nick-repaired (Ion Xpress™ Plus Fragment Library Kit; Ion Xpress™ Barcode Adapters Kit) and then purified again (Agencourt™ AMPure™ XP Kit). The labeled library was then size-selected again using the Pippin Prep™ DNA Size Selection System (Sage Science), aiming for a target-peak size of ~480 bp collected over a specified range. Following sample purification (Agencourt™ AMPure™ XP Kit) the concentration of the unamplified library was determined using qPCR (Ion Library TaqMan® Quantitation Kit). All of the prepared libraries were at an adequate concentration which did not require further amplification. Barcoded libraries were pooled in equimolar amounts of 26 pM to ensure an equal representation of each barcoded library in the sequencing run. The library was then used to prepare enriched, template-positive Ion PGM™ Hi-Q™ Ion Sphere™ Particles (ISPs) using the Ion OneTouch™ 2 System (Ion PGM™ Hi-Q™ OT2 Kit). The recovered template positive ISPs were enriched using the Ion OneTouch™ ES Instrument and Ion OneTouch™ ES Supplies Kit, then loaded onto an Ion 318™ Chip v2 BC and sequenced using the Ion PGM™ Hi-Q™ Sequencing Kit and Ion PGM™ Instrument. The resulting sequencing reads were downloaded from the Torrent Server and analyzed using Geneious R8.1.9 (Biomatters Ltd, New Zealand) with comparison made to *Treponema denticola* ATCC 33520 NCBI Reference Sequences NZ_AGDS00000000.1 and NZ_KB445542.1, and the sequence of our laboratory *T. denticola* 33520 strain. In depth examination of predicted amino acid substitutions due to single nucleotide polymorphisms was performed using NCBI BLASTp followed by COBALT Constraint-based Multiple Alignment Tool (Papadopoulos and Agarwala, 2007).

### Swimming Assay

To compare the motility of *T. denticola* mutants with that of the wild-type ATCC 33520, a swimming assay in semisolid OBGM agar [OBGM supplemented with 0.4% (w/v) Molecular Grade Agarose (Bioline) and 1% (w/v) BD Difco™ gelatin (Bacto Laboratories Pty. Ltd.)] was developed based on previously published motility assays (Lux et al., 2002; Bian et al., 2013). The OBGM agar plates were pre-reduced overnight in an anaerobe chamber. *T. denticola* cells grown to exponential growth phase in OBGM were harvested by centrifugation (4,000 *g*, 6 min, 25°C) and gently suspended in an appropriate volume of deionized water. The bacterial suspension (2 μL; 10^7^ cells) was carefully injected into the semisolid OBGM agar using a pipettor, ensuring that all of the suspension was below the surface. The plates were dried for 20 min at room temperature before they were incubated anaerobically at 37°C for 10 days. Images of *T. denticola* turbid plaques were obtained using a Fujifilm LAS-3000 Imager.

### Cryo-Electron Microscopy (Cryo-EM) and Scanning Electron Microscopy (SEM)

Unwashed *T. denticola* whole cells were prepared for cryo-EM and imaged at the Bio21 Advanced Microscopy Facility, The University of Melbourne, as previously described (Chen et al., 2011) with the following modification. The images were acquired in FEI Tecnai G2 F30 equipped with a FEI Ceta 4 × 4 k CMOS camera and operated at 200 or 300 kV. *T. denticola* and *P. gingivalis* biofilms were prepared and imaged using SEM on a Phillips XL30 Gold-emission scanning 203 electron microscope (Phillips, Eindhoven, The Netherlands) at a voltage of 2 kV as described previously (Mitchell et al., 2010).

### Autoaggregation and Coaggregation Assays

Autoaggregation of *T. denticola* strains and *P. gingivalis* W50 as well as coaggregation between *T. denticola* strains and *P. gingivalis* were evaluated as previously described (Abiko et al., 2014) with some modifications. *T. denticola* and *P. gingivalis* cells were grown to exponential phase before they were harvested by centrifugation (4,000 *g*, 10 min, 25°C). Cells were washed twice with coaggregation buffer (20 mM phosphate buffer, pH 8.0, 1 mM CaCl_2_, 1 mM MgCl_2_, 150 mM NaCl) and suspended in the coaggregation buffer to an A_650_ of 0.5. For the autoaggregation assay, 1 mL of the cell suspension was placed in a cuvette and the height of the bacterial aggregates was monitored for 7 h. For the coaggregation assay, equal volumes of *T. denticola* and *P. gingivalis* cell suspensions were combined in a cuvette to give an A_650_ of 0.5. The height of the bacterial aggregates was monitored for 7 h by measurement with a ruler.

### Quantitative Proteomic Analyses

Whole cell samples were analyzed by sodium dodecyl sulfate polyacrylamide gel electrophoresis (SDS-PAGE) on a 10% (w/v) NuPAGE® Novex® Bis-Tris precast mini-gel. The gel was run at 150 V for ~10 min and the whole gel band from well to dye front was excised from the SimplyBlue™ SafeStain stained SDS-PAGE gel. In-gel digestion was performed after reduction with 10 mM DTT and alkylation with 50 mM iodoacetamide using sequencing-grade-modified trypsin (Promega) overnight at 37°C, as previously published (Mortz et al., 2001). The digestion was stopped by the addition of TFA to a final concentration of 0.1% (v/v). The fluid was transferred to a new tube for analysis by LC-MS/MS as previously described (Glew et al., 2017).

Raw MS files were analyzed by MaxQuant (Ver 1.5.3.30) (Cox et al., 2014) using label-free quantification (LFQ) searching against the whole *T. denticola* ATCC 35405 protein database containing a total of 2,786 protein sequences obtained from the Comprehensive Microbial Resource Website (cmr.jcvi.org). The default parameters were used for LFQ. Data analysis was performed in Excel, using the MaxQuant output file “proteinGroups.txt.” Any contaminating eukaryotic proteins, such as human keratins or serum proteins from the growth media were removed from the list of identified proteins. LFQ intensity values were used to produce ratios of paired samples and the geometric mean of these ratios was calculated. Statistical analysis was performed using paired, two-tailed Student's *t*-tests. The abundance of a protein was considered to have significantly changed between two samples if there was ≥1.5- or ≤0.67-fold change in the geometric mean and a *p-*value of <0.05.

### Static Biofilm Assay

Static biofilm assays were carried out essentially as previously described (Dashper et al., 2014). Briefly, *T. denticola* and *P. gingivalis* cells were grown to exponential phase and diluted with fresh pre-reduced OBGM to A_650_ of 0.15 where necessary. For monospecies biofilms, 2 mL of each cell suspension was aliquoted into each well of a CELLSTAR® 12-well flat-well plate (Greiner, Sigma-Aldrich). For dual-species biofilms, equal volumes of *T. denticola* and *P. gingivalis* cell suspensions were aliquoted into each well of the 12-well flat-well plate and mixed. After inoculation, the plates were incubated at 37°C anaerobically for 1 h, sealed with a Greiner multiwell plate sealer (Sigma-Aldrich) and further incubated at 37°C anaerobically for 5 days. The attached biofilms were quantified by crystal violet staining. Following incubation, the medium in each well was decanted and each well was gently rinsed with 2.1 mL of deionized water. The plates were air-dried and the biofilms were stained with 0.1% (v/v) crystal violet for 30 min at RT, then rinsed twice with 2.5 mL of deionized water. The plates were air-dried and destained with 2.1 mL of 99% (v/v) ethanol and the absorbance of each well at 540 nm (A_540_) was measured using a Wallac VICTOR3™ 1420 Multilabel Counter (PerkinElmer, USA). A Kruskal-Wallis rank sum test was conducted with the Conover-Imam test for the statistical analysis of the biofilm data (Kruskal and Wallis, 1952; Conover and Iman, 1979).

## Results

### *T. denticola* Motility Mutants

*T. denticola* ATCC 33520 motility mutants were generated via electroporation as previously described, with some modifications (Capone et al., 2007). Significantly, *T. denticola* cells were washed twice instead of thrice under strictly anaerobic conditions to improve transformation efficiency. Positive Erm^R^ clones resulting from double-crossover homologous recombination following electroporation with linearized pHN-Δ*flgE* and pHN-Δ*motB* were verified by PCR using the primer pairs 5′flgE-F/3′flgE-R and 5′motB-F/3′motB-R, respectively. The modified transformation protocol used in this study resulted in consistently achieving 98–100% true positive transformants and eliminated the complication of spontaneous Erm resistant colonies commonly reported for earlier protocols (Bian et al., 2012). Validated deletant strains of *T. denticola* Δ*flgE* and Δ*motB* were used for further analyses.

Genomic sequencing was performed to confirm the veracity of the mutants [NCBI Bioproject accession number PRJNA561478]. Both mutants had the appropriate target genes deleted from their genomes. Between the two mutants, a total of four different loci were affected by single nucleotide polymorphisms (SNPs), with one found in both mutants (Supplementary Table 2). None of the SNPs were found in the *T. denticola* 33520 laboratory strain. These SNPs resulted in amino acid substitutions in two motility related proteins and were predicted to have no effect on protein structure or function as the substituted amino acids were all found in nature in at least one other *T. denticola* strain, as determined using COBALT (data not shown). The only locus which was subject to a frame shift occurred in Δ*flgE* whereby the insertion of a single C in hypothetical protein HMPREF9722_RS03985 resulted in Δ*flgE* missing the final 5% of this protein. Interestingly 14% of all sequencing reads at this locus in the WT also had this identical C insertion suggesting that this mutation may possibly be a common mutation emerging in WT. This hypothetical protein was detected in ΔFlgE using a proteomics approach (see below).

### Phenotypic Characterization of *T. denticola* Wild-Type, Δ*flgE* and Δ*motB*

**Figure 2:**
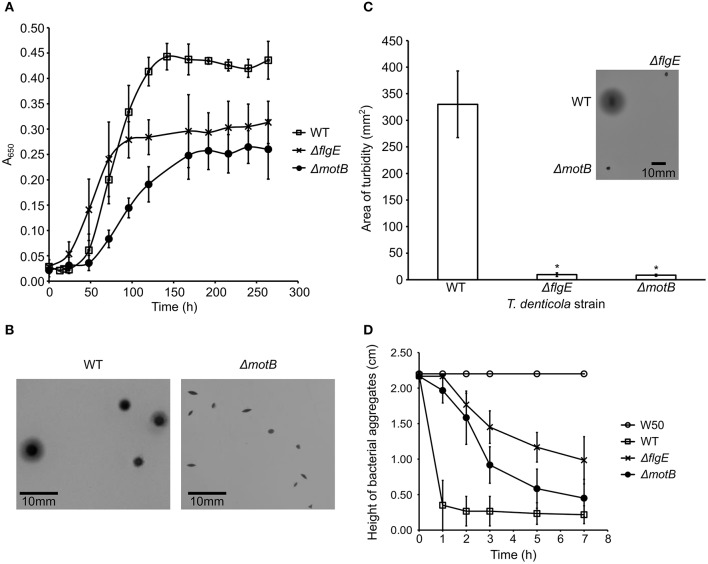
Characterization of *T. denticola* wild-type, Δ*flgE*, Δ*motB*. **(A)** Growth of *T. denticola* wild-type and mutants. *T. denticola* cultures at stationary phase were inoculated into fresh OBGM medium at *t* = 0. A_650_ of the cultures was measured at 6–24 h intervals for 264 h. The mean generation time was calculated based on the rate of change of A_650_ during the exponential growth phase of cultures (*N* = 3). The mean generation times were statistically analyzed using a one way ANOVA with both mutants Δ*motB* and Δ*flgE* significantly different (*p* < 0.05) to WT. **(B)** Colony morphologies of *T. denticola* wild-type and Δ*motB*. *T. denticola* cells were inoculated into OBGM agar [0.8% (w/v) agarose] and poured into petri dishes which were then incubated anaerobically for 2 weeks. The colonies formed by Δ*flgE* had a similar morphology to the colonies of Δ*motB*. **(C)** Swimming assay of *T. denticola* wild-type and mutants. *T. denticola* cells harvested at exponential phase were spotted on semisolid OBGM agar [0.4% (w/v) agarose and 1% (w/v) gelatin] and incubated anaerobically for 10 days before the area of turbid plaque was measured. The data are presented as means plus standard deviations (*N* = 29) and were analyzed by Students' *T*-test. Values that were significantly different (*p* < 0.05) from the value for *T. denticola* wild-type are indicated by an asterisk (^*^). A representative image of the swimming assays of *T. denticola* wild-type, Δ*flgE*, Δ*motB* after 10 days of anaerobic incubation is shown. **(D)** Autoaggregation of *P. gingivalis* W50, *T. denticola* wild-type and mutants. *T. denticola* and *P. gingivalis* cells harvested at exponential phase were washed twice with coaggregation buffer and adjusted to A_650_ of 0.5. The height of bacterial aggregates was monitored for 7 h. The data are presented as means and standard deviations (*N* = 3).

The planktonic growth of *T. denticola* Δ*motB* and Δ*flgE* mutants was impaired compared with the wild-type. The mutants had a lower maximum cell density at stationary phase and a longer mean generation time than wild-type (Figure 2A). The mean generation times for wild-type, Δ*flgE* and Δ*motB* were 14 ± 3, 22 ± 3, and 24 ± 5 h, respectively. When grown on agar plates, the colonies of Δ*flgE* (data not shown) and Δ*motB* were small, dense, and pinpoint-shaped. The edges of the colonies were defined instead of diffusing outward like that of the wild-type (Figure 2B). Swimming assay analysis revealed that the mutants were non-motile as they were unable to swim outward from the point of inoculation and form a turbid plaque like those of the wild-type (Figure 2C). Autoaggregation assays were carried out to examine the ability of *P. gingivalis* W50, *T. denticola* wild-type and mutants to bind to themselves. *P. gingivalis* did not autoaggregate in this assay whereas *T. denticola* wild-type autoaggregated at the highest rate, followed by Δ*motB* and finally Δ*flgE* (Figure 2D).

### Cryo-Electron Microscopy

**Figure 3:**
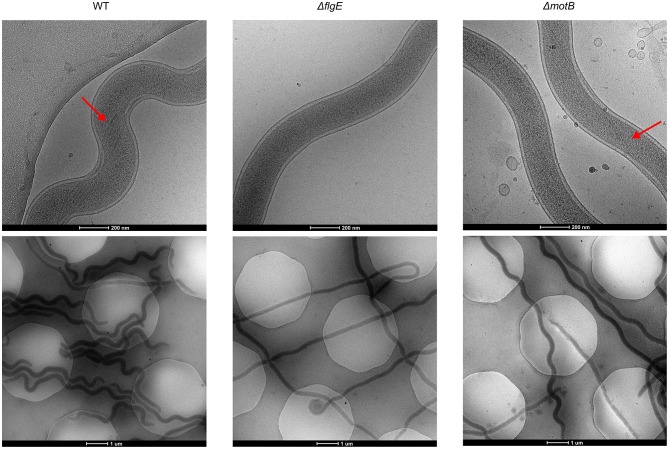
Representative cryo-Electron Microscopy images of *T. denticola* wild-type, Δ*flgE*, and Δ*motB. T. denticola* cells were grown to exponential phase and viewed directly under cryo-EM. Red arrows point to the periplasmic flagella.

*T. denticola* wild-type and mutant cells in planktonic exponential growth phase were imaged with cryo-electron microscopy to examine periplasmic flagella and to determine cellular morphology (Figure 3). Four or five periplasmic flagella were distinguishable in *T. denticola* wild-type cells and no periplasmic flagella were observed in Δ*flgE*. Only two or three periplasmic flagella were distinguishable in Δ*motB* and not all cells or cell segments of Δ*motB* showed visible flagella. *T. denticola* wild-type cells adopted an irregular twisted morphology with both planar and helical regions. The Δ*motB* cells appeared to have less spirality than the wild-type while many cells or cell segments of Δ*flgE* had limited or no spirality and appeared rod-shaped.

### Quantitative Proteomics of Δ*flgE* and Δ*motB*

In order to identify proteins that changed in abundance in the motility mutants relative to the wild-type, whole cell lysates were examined using quantitative proteomics. The proteins FlgE (TDE2768) and MotB (TDE2765) were found in wild-type but not in the Δ*flgE* and Δ*motB* mutants, respectively, further confirming the deletion of these genes in the mutants.

There were 64 and 326 proteins that changed in abundance in Δ*flgE* and Δ*motB*, respectively, relative to the wild-type (Supplementary Tables 3–5). Proteins that significantly changed in abundance were sorted into functional categories on the basis of clusters of orthologous groups (COGs) (Tatusov et al., 1997, 2003). Of the 64 proteins in Δ*flgE* that changed in abundance, 42 significantly decreased and 22 significantly increased. COG category N (cell motility) had the highest number of proteins (19%) (Table 1) that changed in abundance in Δ*flgE* indicating the wider effects of this mutation on flagella and motility. Of the 326 proteins in Δ*motB* that changed in abundance, 229 significantly decreased and 97 significantly increased. COGs related to metabolism contained the highest number of proteins (28%), followed by cellular processes and signaling (25%) and information storage and processing (10%) (Table 1).

**Table 1:** Proteins significantly changed in abundance in *T. denticola* Δ*flgE* and Δ*motB* relative to wild-type, grouped by COG category.

	**COG^**a**^**	**Number of proteins**	
		**Δ*flgE***	**Δ*motB***
Information storage and processing	J	3	18
	K	1	5
	L	2	9
Cellular processes and signaling	D	1	4
	M	3	14
	N	12	14
	O	3	12
	T	1	23
	U	2	9
	V	–	4
Metabolism	C	5	12
	E	2	22
	F	3	10
	G	1	16
	H	1	7
	I	2	6
	P	3	17
	Q	–	2
Poorly or not characterized	R	–	8
	S	17	94
	N/A	2	20
	Total	64	326

a *One-letter abbreviations for the functional COG categories: J, translation, ribosomal structure and biogenesis; K, transcription; L, replication, recombination and repair; D, cell cycle control, cell division, chromosome partitioning; V, defense mechanisms; T, signal transduction mechanisms; M, cell wall/membrane/envelope biogenesis; N, cell motility; U, intracellular trafficking, secretion, and vesicular transport; O, post-translational modification, protein turnover, chaperones; C, energy production and conversion; G, carbohydrate transport and metabolism; E, amino acid transport and metabolism; F, nucleotide transport and metabolism; H, coenzyme transport and metabolism; I, lipid transport and metabolism; P, inorganic ion transport and metabolism; Q, secondary metabolites biosynthesis, transport and catabolism; R, general function prediction only; S, function unknown*.

Thirty-five proteins changed in abundance in both mutants with the largest number being in COG category N (Table 2). All of these proteins, including those expressed from the *fla* operon (TDE2764-TDE2768), FlaB (TDE1004), FlaA (TDE1408, TDE1409, TDE1712), and a flagellar hook-associated protein FlgL (TDE2353), significantly decreased in abundance in both mutants, except TDE0119 (FliS) which decreased in abundance in Δ*motB* but increased in abundance in Δ*flgE* (Table 3).

**Table 2:** Proteins significantly changed in abundance in both *T. denticola* Δ*flgE* and Δ*motB* or Δ*motB* only, grouped by COG category.

	**COG^**a**^**	**Number of proteins**		
		**Changed in abundance in both mutants**	**Increased in abundance in Δ*motB* only**	**Decreased in abundance in Δ*motB* only**
Information storage and processing	J	2	12	4
	K	1	1	3
	L	2	2	5
Cellular processes and signaling	D	–	3	1
	M	–	2	12
	N	10	–	5
	O	1	2	9
	T	1	5	17
	U	1	3	3
	V	–	1	3
Metabolism	C	1	6	5
	E	1	6	15
	F	2	4	4
	G	–	5	11
	H	–	3	4
	I	–	–	6
	P	3	1	14
	Q	–	–	2
Poorly or not characterized	R	–	1	6
	S	5	23	63
	N/A	5	6	13
	Total	35	86	205

a *One-letter abbreviations for the functional COG categories: J, translation, ribosomal structure and biogenesis; K, transcription; L, replication, recombination and repair; D, cell cycle control, cell division, chromosome partitioning; V, defense mechanisms; T, signal transduction mechanisms; M, cell wall/membrane/envelope biogenesis; N, cell motility; U, intracellular trafficking, secretion, and vesicular transport; O, post-translational modification, protein turnover, chaperones; C, energy production and conversion; G, carbohydrate transport and metabolism; E, amino acid transport and metabolism; F, nucleotide transport and metabolism; H, coenzyme transport and metabolism; I, lipid transport and metabolism; P, inorganic ion transport and metabolism; Q, secondary metabolites biosynthesis, transport and catabolism; R, general function prediction only; S, function unknown*.

**Table 3:** Proteins significantly changed in abundance in both Δ*motB* and Δ*flgE* mutants relative to wild-type (ratio ≥1.5 and ≤0.67, *p* < 0.05).

**Locus tag**	**Protein description**	**Wild-type abundance^**a**^**	**Δ*motB* ratio^**b**^**	**Δ*flgE* ratio^**b**^**	**COG^**c**^**
TDE0114	Iron-dependent transcriptional regulator	3.46E+07	0.59	0.55	K
TDE0119	Flagellar protein FliS	1.49E+06	0.00	2.71	N
TDE0383	Hypothetical protein	5.62E+06	0.00	0.00	S
TDE0423	Hypothetical protein	1.85E+07	0.27	0.53	–
TDE0463	Purine nucleoside phosphorylase (DeoD)	5.41E+07	0.55	0.62	F
TDE0501	Hypothetical protein	9.24E+06	0.00	0.50	–
TDE0689	5-Methylthioribose kinase	1.51E+06	0.00	0.00	S
TDE0758	Iron compound ABC transporter, periplasmic iron compound-binding protein, putative	6.66E+06	4.14	0.23	P
TDE0781	Ribosomal protein S8 (rpsH)	4.62E+07	1.61	0.65	J
TDE0984	Oligopeptide/dipeptide ABC transporter, permease protein, putative	5.71E+06	8.79	0.00	P
TDE0985	Oligopeptide/dipeptide ABC transporter, periplasmic peptide-binding protein, putative	3.22E+08	5.20	0.33	E
TDE0986	Oligopeptide/dipeptide ABC transporter, ATP-binding protein	2.92E+06	12.92	0.00	P
TDE1004	Flagellar filament core protein (FlaB)	5.28E+08	0.24	0.01	N
TDE1208	DNA topoisomerase I (TopA)	6.03E+07	1.93	1.79	L
TDE1234	Hypothetical protein	3.64E+05	3.81	1.51	–
TDE1318	Hypothetical protein	7.60E+05	0.00	0.00	–
TDE1408	Flagellar filament outer layer protein (FlaA)	5.92E+08	0.29	0.04	N
TDE1409	Flagellar filament outer layer protein (FlaA)	5.59E+08	0.28	0.04	N
TDE1483	Conserved hypothetical protein	1.28E+08	1.52	0.30	S
TDE1712	Flagellar filament outer layer protein (FlaA)	1.85E+09	0.18	0.02	N
TDE1727	Conserved hypothetical protein	1.03E+08	0.54	0.46	O
TDE1754	Desulfoferrodoxin/neelaredoxin	6.80E+07	2.71	0.54	C
TDE1919	Conserved domain protein	1.64E+07	0.63	1.90	S
TDE2043	Signal recognition particle-docking protein FtsY (ftsY)	5.55E+06	0.00	0.64	U
TDE2085	Amino acid kinase family protein	7.21E+07	0.46	1.55	F
TDE2087	Translation initiation factor IF-1 (infA)	4.30E+07	1.68	0.61	J
TDE2302	HD domain protein	1.09E+07	0.00	0.64	T
TDE2353	Flagellar hook-associated protein (FlgL)	1.83E+06	0.00	0.59	N
TDE2611	Conserved hypothetical protein	2.62E+06	0.32	1.52	S
TDE2721	Helicase domain protein	3.38E+06	2.70	0.67	L
TDE2764	Flagellar protein FliL	6.46E+07	0.12	0.50	N
TDE2765	Flagellar motor rotation protein B (MotB)	3.44E+07	0.00	0.35	N
TDE2766	Motility protein A (MotA)	3.46E+07	0.42	0.44	N
TDE2768	Flagellar hook protein FlgE	4.39E+07	0.33	0.00	N
TDE2779	Hypothetical protein	5.95E+07	0.10	0.31	–

*Shading indicates proteins predicted to be organized in an operon*.

a *The abundance of each protein in the wild-type T. denticola wild-type was calculated from the average IBAQ intensity from three replicates*.

b *Geometric mean of ratios, from three replicates, produced from the LFQ intensity of protein in mutant relative to that of protein in wild-type. Ratio of ≥1.5 indicates that the protein had increased in abundance in mutant relative to wild-type and ratio of ≤0.67 indicates that the protein had decreased in abundance in mutant relative to wild-type. Zero ratio indicates that the protein was identified in ATCC 33520 but not in the mutant*.

c *One-letter abbreviations for the functional COG categories: J, translation, ribosomal structure and biogenesis; K, transcription; L, replication, recombination and repair; D, cell cycle control, cell division, chromosome partitioning; V, defense mechanisms; T, signal transduction mechanisms; M, cell wall/membrane/envelope biogenesis; N, cell motility; U, intracellular trafficking, secretion, and vesicular transport; O, post-translational modification, protein turnover, chaperones; C, energy production and conversion; G, carbohydrate transport and metabolism; E, amino acid transport and metabolism; F, nucleotide transport and metabolism; H, coenzyme transport and metabolism; I, lipid transport and metabolism; P, inorganic ion transport and metabolism; Q, secondary metabolites biosynthesis, transport and catabolism; R, general function prediction only; S, function unknown*.

Of the proteins that increased in abundance in Δ*motB* only, COG category J (translation, ribosomal structure and biogenesis) contained the highest number of proteins (Table 2). These proteins include several aminoacyl-tRNA synthetases, such as valyl-tRNA synthetase (TDE1364), tryptophanyl-tRNA synthetase (TDE1588), and phenylalanyl-tRNA synthetase (TDE1927), suggesting an increased usage of the corresponding amino acids valine, tryptophan and phenylalanine in Δ*motB*. There was also an increase in the abundance of recombination protein A (RecA; TDE0872), DNA topoisomerase I (TopA; TDE1208), transcription termination factor Rho (Rho; TDE1503) and a rod-shaped determining protein MreB (TDE1349). The metabolic enzymes desulfoferrodoxin/neelaredoxin (TDE1754), FAD-dependent oxidoreductase (TDE2643), and Na^+^-translocating NADH-quinone reductase E subunit (NqrE; TDE0834) were also among the proteins that increased in abundance in Δ*motB*. Two methyl-accepting chemotaxis proteins (MCPs; TDE1009 and TDE2549), two metallo-beta-lactamase family proteins (TDE1444 and TDE1541) and several ABC transporter proteins (TDE0758, TDE0983–TDE0987) also increased in abundance in Δ*motB*.

Of the proteins that decreased in abundance in Δ*motB* only, COG category T (signal transduction mechanisms) contained the highest number of proteins (Table 2). FleN (TDE2685), an MCP (TDE2783), two chemotaxis proteins (CheX and CheY) and two penicillin binding proteins (PBPs; TDE1314 and TDE1352) were among the proteins that decreased in abundance. *T. denticola* virulence factors including Msp (TDE0405), hemolysin (TDE1669), and the glycine reductase complex proteins GrdD (TDE0239) and GrdE2 (TDE2120) also decreased in abundance.

### Coaggregation Assay of *T. denticola* Strains With *P. gingivalis*

**Figure 4:**
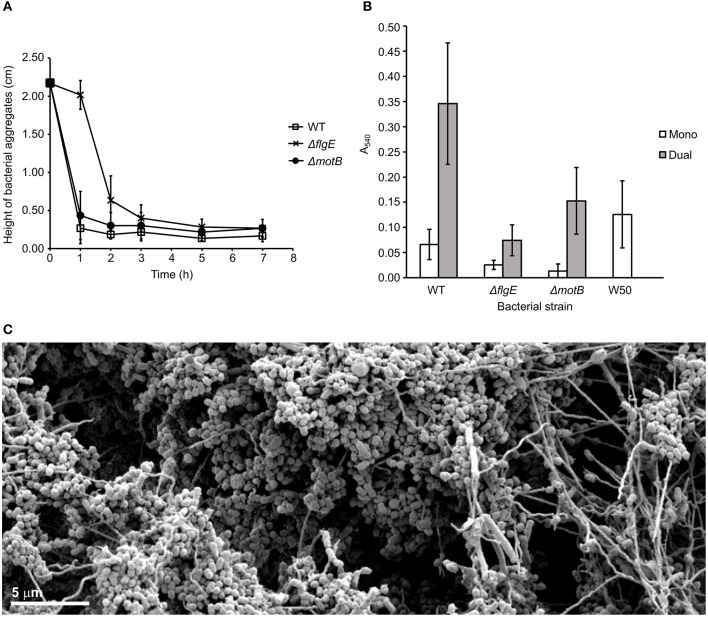
Coaggregation and biofilm formation of *T. denticola* wild-type, Δ*flgE*, Δ*motB* with *P. gingivalis* W50. **(A)** Coaggregation of *T. denticola* wild-type and mutants with *P. gingivalis* W50. *T. denticola* ATCC 33520 and *P. gingivalis* cells at exponential growth phase were harvested, washed twice with coaggregation buffer and adjusted to A_650_ of 0.5 with the coaggregation buffer. Equal volumes of *T. denticola* and *P. gingivalis* cell suspensions were combined. The height of bacterial aggregates was monitored for 7 h. The data are presented as means plus standard deviations (*N* = 3). **(B)** Monospecies and dual-species static biofilms of *T. denticola* ATCC 33520 wild-type, Δ*flgE*, Δ*motB* with *P. gingivalis* W50. *T. denticola* and *P. gingivalis* cells were grown to exponential phase, diluted to A_650_ of 0.15 and grown anaerobically for 5 days in a 12-well plate, either in a monospecies or dual-species culture. The resultant biofilm was stained with crystal violet and the total biomass determined spectrophotometrically. The data are presented as means and standard deviations (*N* = 23–27) and were analyzed using a Kruskal-Wallis with Conover-Imam test. All values were significantly different (*p* < 0.05) except between the following pairs: Δ*motB* (mono); wild-type (mono) and Δ*flgE* (dual); as well as wild-type (dual). **(C)** SEM image of a biofilm containing *T. denticola* wild type ATCC 35405 (the long thin spirochaete) and *P. gingivalis* W50 (the grape-like coccobacillus). The image shows the motile *T. denticola* facilitating expansion of the biofilm microcolonies by forming bridge structures while carrying *P. gingivalis*.

*T. denticola* Δ*motB* coaggregated with *P. gingivalis* at the same rate as wild-type but Δ*flgE* coaggregated with *P. gingivalis* at a slower rate than the wild-type (Figure 4A).

### Biofilm Assay of *T. denticola* Strains With *P. gingivalis*

A biofilm assay was used to determine the ability of *T. denticola* wild-type and motility mutants to form monospecies biofilms and dual-species biofilms with *P. gingivalis* W50. The *T. denticola* motility mutants Δ*flgE* and Δ*motB* formed monospecies biofilms with significantly less biomass than those formed by the wild-type (Figure 4B). A synergy was observed between *T. denticola* wild-type and *P. gingivalis* in dual-species biofilm formation, as demonstrated by an ~2-fold higher biomass of the dual-species biofilms than the sum of the monospecies biofilms (Figure 4B). The motility of wild type *T. denticola* facilitated expansion of the biofilm through the formation of bridges and transport of *P. gingivalis* (Figure 4C; Zhu et al., 2013). *T. denticola* Δ*flgE* showed a considerably reduced ability to form dual-species biofilms with *P. gingivalis* compared with the wild-type, with a 5-fold reduction in biomass. Interestingly, Δ*flgE* dual-species biofilms were smaller than *P. gingivalis* monospecies biofilms (*p* < 0.05). Δ*motB* formed biofilms with *P. gingivalis* that had a 2-fold lower biomass than those formed with the wild-type (Figure 4B).

## Discussion

Although not generally referred to as classic virulence factors in most human pathogens, motility and chemotaxis are undoubtedly important in bacterial-host interactions and disease progression (Dashper et al., 2011). The combination of a chemotaxis system and motility enables efficient nutrient acquisition, avoidance of toxic substances and translocation to optimal colonization sites by bacteria. It is thus important in the survival and proliferation of bacteria, especially for nutritionally fastidious organisms, such as the oral treponemes. Approximately 5–6% of the genome of sequenced treponemes is dedicated to motility and chemotaxis (Seshadri et al., 2004). The motility of *T. denticola* is dependent on its periplasmic flagella which, unlike the exposed flagella of most motile bacteria, are located in the periplasmic space between the outer and cytoplasmic membranes of the cells (Chi et al., 1999). *T. denticola* Δ*flgE* and Δ*motB* mutants lacking specific genes involved in the motility machinery were generated to investigate the importance of *T. denticola* periplasmic flagella and/or motility in synergistic biofilm formation with *P. gingivalis*.

Traditionally the roles of gene products in bacterial phenotypes and behaviors are determined by the specific inactivation or deletion of the gene under investigation. In this study we confirmed the specific nature of gene deletion by sequencing the genomes of the mutants. The genome of ΔMotB contained no predicted effects on protein structure other than the absence of an ORF encoding *motB*. The genome of ΔFlgE contained a deletion of *flgE* ORF and only one other change causing a truncation of the last 5% of hypothetical protein, HMPREF9722_RS03985. The phenotypic effects of this truncation remain unknown, nevertheless proteomics data revealed that this protein is present in ΔFlgE. However, quantitative proteomic analyses of the mutants revealed that the specific deletion of the *motB* gene from the *T. denticola* genome had wide ranging effects on the overall protein abundance profiles of the mutants.

Among the 35 proteins that changed in abundance in both mutants, 10 were related to flagella and motility. All of them were reduced in abundance in both mutants, except FliS which had increased in abundance in Δ*flgE*. Completion of the hook-basal body structure serves as a checkpoint for transcriptional regulation of flagellum synthesis (Hughes et al., 1993). Therefore, the deletion of *motB* and *flgE* may have negatively regulated the transcription of genes from within the *fla* operon where *motB* and *flgE* are located as well as those that are not located in the *fla* operon. These included *fliS, flgL, flaA*, and *flaB* genes. It is also possible that the regulation was mediated at the post-transcriptional level as was seen in the reduced abundance of FlaA and FlaB proteins in a *Borrelia burgdorferi* Δ*flgE* mutant (Sal et al., 2008). Moreover, the presence of MotB might also act as a checkpoint for the expression of chemotaxis genes as several chemotaxis-related proteins were differentially regulated in Δ*motB*. Together, these results suggest that flagellum synthesis and the expression of chemotaxis and motility genes utilizes a finely regulated process where there is a series of transcriptional and/or post-transcriptional controls.

Both Δ*motB* and Δ*flgE* were non-motile. Although *T. denticola* ATCC 33520 is commonly observed to have four periplasmic flagella (Izard et al., 2008), in this study up to five periplasmic flagella were occasionally observed (Figure 3). As expected, the Δ*flgE* mutant was deficient in periplasmic flagella as it lacks the flagellar hook protein, FlgE, necessary for flagella assembly (Li et al., 1996). Periplasmic flagella were observed in Δ*motB* mutants, but at a lower number than the wild-type and not all cell segments of the mutants showed visible flagella (Figure 3). The reduction in periplasmic flagella number in Δ*motB* is consistent with the quantitative proteomic results which showed a reduction in the abundance of the flagellar filament proteins, FlaA and FlaB, in this mutant relative to wild-type. Furthermore, FleN, a protein involved in the regulation of flagella number in *Pseudomonas aeruginosa* (Dasgupta et al., 2000), was also decreased in abundance in Δ*motB*. A *T. denticola* non-motile mutant lacking FliG, a flagellar motor protein, had a markedly decreased number of flagellar filaments and the flagellar filaments were usually shorter in length than those of the wild-type (Slivienski-Gebhardt et al., 2004). In addition, there was a reduction in the FlaA and FlaB proteins in the Δ*fliG* mutant (Slivienski-Gebhardt et al., 2004). The similar phenotype of the Δ*fliG* mutant with Δ*motB* suggested the importance of the flagellar motor in the complete assembly of *T. denticola* periplasmic flagella.

The cellular morphologies of Δ*motB* and Δ*flgE* were different to that of the wild-type. The *T. denticola* periplasmic flagella contribute to the irregular twisted morphology of the bacterium, which is the predominant form adopted by the cells in planktonic exponential phase, with the other being the regular right-handed helical form (Ruby et al., 1997). *T. denticola* wild-type cells of both strains 35405 and 33520 with the outer membrane removed and *T. denticola* mutants lacking periplasmic flagella lost their irregular morphology and adopted a helical form (Ruby et al., 1997). In this study, an irregular morphology was observed in *T. denticola* wild-type cells. However, unlike the previous study, the Δ*flgE* mutant lacking periplasmic flagella adopted a rod shape instead of a helical shape (Figure 3). This result is more similar to that observed in *B. burgdorferi* where a Δ*flaB* mutant deficient in periplasmic flagella lost its flat-wave morphology and adopted a rod shape (Motaleb et al., 2000). The reason for the discrepancy in the observed morphology of Δ*flgE* mutant in this study with the previous study is unclear since the same strain of *T. denticola* harvested at the same growth phase was used and both mutants were deficient in periplasmic flagella. Differences in the growth media and the temperature of incubation between studies were evident but it is uncertain how these would affect the cell morphology. The most likely reason for these discrepancies may lie in the specific mutation of the genomes. In the previous study the *T. denticola* 33520 aflagellated mutant was a spontaneous occurring mutant that remained genetically uncharacterized. Furthermore, the *T. denticola* 35405 specific FlgE- mutant which was created by deletion of *flgE* from the chromosome by Erm cassette insertion did not involve reintroduction of the promoter sequence downstream of the Erm cassette in the genome as has been done in this study. In the present study we have used RT-PCR (results not shown) to show that transcription of the six operon genes (*motA, motB, fliL, fliM, fliY*, and *fliP*) downstream of deleted *flgE* remain unaffected by the introduction of *ermAM*. Furthermore, the phenotypic effect of the possible truncation of the C-terminus of hypothetical protein HMPREF9722_RS03985 in ΔFlgE in the present study remains unknown.

When compared to wild-type, Δ*motB* appeared less spiral and more rod-like which could be caused by the observed reduction in the periplasmic flagella number. Alternatively, it might be a result of the change in abundance of proteins that are involved in the morphology of bacteria, as observed in the spirochete *Leptospira* (Slamti et al., 2011). These proteins include the penicillin binding proteins (PBPs) and rod shape-determining cytoskeleton protein MreB, which likely controls cell morphology through their interactions with the peptidoglycan layer (Divakaruni et al., 2007). The PBPs were reduced in abundance while MreB increased in abundance in the Δ*motB* mutant, suggesting a switch in the mechanism of cell shape maintenance. Interestingly, the cellular localization of the MreB cytoskeleton in *Bacillus subtilis* requires a transmembrane ion motive force (IMF); disruption of the IMF resulted in a rapid delocalization and loss of helicity of the MreB cytoskeleton (Strahl and Hamoen, 2010). As the IMF is predicted to be disrupted in Δ*motB*, it is possible that the cells increased the production of MreB in order to compensate for the delocalization of MreB. Furthermore, the altered cellular morphology could also be caused by a lack of flagellar rotation. In *B. burgdorferi*, flagellar rotation is required for the flat-wave morphology of the bacterium and the deletion of *motB* in *B. burgdorferi* caused part of the cells to be rod shaped (Sultan et al., 2015). Overall, these results suggest that the presence of properly assembled, functional periplasmic flagella is an important determinant of the characteristic spiral morphology of *T. denticola*.

The loss of motility impaired the growth of Δ*motB* and Δ*flgE*. One of the reasons for this could be a lack of nutrient accessibility as a result of the loss of motility. In addition, the generation of the transmembrane IMF through the ion channel complex formed by MotA and MotB may be an important source of energy for *T. denticola* growth. The lack of MotB, especially the plug segment required for suppression of undesirable flow through the MotA/B ion channel complex (Hosking et al., 2006; Morimoto et al., 2010) may have resulted in transmembrane ion leakage that negatively affected energy and growth. There was a significant increase in the abundance of an oligopeptide/dipeptide ABC transporter made up of proteins TDE0983–TDE0987 in Δ*motB*, possibly to compensate for a reduction in oligopeptides or dipeptides usually transported into the cell by IMF-driven systems. This transport system, which relies on ATP hydrolysis to drive the transport of substrates across the cell membrane, would be more inefficient than the IMF-driven system and would result in Δ*motB* growing slower than the wild-type, as reflected in the growth studies.

In addition, the disruption of MotB increased the abundance of a number of proteins involved in the bacterial stress response, including desulfoferrodoxin/neelaredoxin (TDE1754), RecA (TDE0872), DNA topoisomerase I (TopA; TDE1208), and transcription termination factor Rho (TDE1503) (Brennan et al., 1987; Jovanovic et al., 2000; Italiani et al., 2002; Liu et al., 2011). Furthermore, two aminoacyl-tRNA synthetases, valyl-tRNA synthetase (TDE1364) and phenylalanyl-tRNA synthetase (TDE1927), involved in the editing of misacylated tRNAs (Ling et al., 2009) and proposed to be critical for bacterial stress responses and survival (Bullwinkle and Ibba, 2016) were also increased in abundance. The observed reduction in the abundance of the PBPs, which are essential for the synthesis of the peptidoglycan layer of the cell (Sauvage et al., 2008), suggested that Δ*motB* were under peptidoglycan stress. The increased abundance of the metallo-beta-lactamase family proteins (TDE1444 and TDE1541) is likely to be a response to peptidoglycan stress as the cells may perceive the reduction in peptidoglycan synthesis as a result of PBPs inhibition and thus produce more metallo-beta-lactamases, the enzymes that catalyze the hydrolysis of beta-lactam antibiotics that inhibit PBPs (Palzkill, 2013). Overall, these results show the importance of the MotA/B proton channel complex in *T. denticola*.

The Na^+^-translocating NADH/quinone reductase E subunit (NqrE; TDE0834) increased in abundance in the Δ*motB* mutant. NqrE is part of the Na^+^-translocating NADH/quinone oxidoreductase (Na^+^-NQR) respiratory complex found in prokaryotes (Barquera, 2014). Na^+^-NQR translocates sodium ions across the membrane, generating an electrochemical Na^+^ gradient which energizes important functions in the cell, including rotation of the Na^+^-dependent flagella for motility (Barquera, 2014). The membrane-embedded stators, Mot complexes, harness energy of either transmembrane H^+^ or Na^+^ ion gradients to power flagellar rotation. There are two distinct types of Mot stators with different ion specificities. In *B. subtilis*, MotA and MotB constitute the H^+^-coupled Mot while MotP and MotS constitute a Na^+^-coupled Mot (Ito et al., 2005; Terahara et al., 2008). In *Vibrio*, the force-generating unit of the Na^+^-driven flagellar motor is composed of four components: PomA, PomB, MotX, and MotY (Li et al., 2011). The use of Na^+^ ion gradients is often associated with elevated pH and sodium concentrations (Mulkidjanian et al., 2008).

The increase in abundance of TDE0834 (NqrE) in Δ*motB* may suggest that the mutant has switched to the usage of Na^+^ as a coupling ion for flagellar motor rotation, instead of H^+^, as a result of IMF disruption. The switching to the use of Na^+^ instead of, or in addition to, H^+^ for flagellar motor rotation may be an adaptation of *T. denticola* to the increase in pH and salinity in an inflamed periodontal pocket during chronic periodontitis. It has been reported that the progression of periodontitis is associated with a rise in pH of the gingival sulcus (Zilm et al., 2010; Barros et al., 2016). Maintaining high levels of IMF would become increasingly difficult for *T. denticola* as the external concentration of H^+^ ions become lower. In addition, the concentration of Na^+^ ions in the periodontal pocket increases as a result of bleeding and tissue inflammation (Barros et al., 2016). Switching to the use of Na^+^ ions may thus be more advantageous for *T. denticola* in this relatively alkaline and sodium-rich environment. This hypothesis is supported by a study which shows that the MotA/B of *Bacillus clausii*, an alkaliphilic bacterium, is bifunctional with respect to ion-coupling capacity in that it couples motility to sodium at high pH (pH > 8.5) but uses protons at lower pH (pH < 8.5) (Terahara et al., 2008). *B. clausii* MotA/B increases its use of sodium as the pH becomes increasingly alkaline (Terahara et al., 2008) and presumably the IMF is getting smaller. The observed increase in the component of Na^+^-NQR in Δ*motB* with the predicted disruption in IMF may thus suggest that the ion-coupling pattern of *T. denticola* MotA/B changes as the relative magnitudes of the transmembrane sodium and proton motive forces change.

In this study, *T. denticola* formed detectable monospecies biofilms. Although generally regarded as a poor biofilm former (Davey, 2006; Biyikoglu et al., 2012), *P. gingivalis* W50 was also able to form a biofilm (Figure 4B). Compared to the wild-type, both *T. denticola* motility mutants had reduced abilities to form a mono-species biofilm. Together with the autoaggregation results (Figure 2D), this indicated the importance of motility in the binding of *T. denticola* cells for monospecies biofilm formation under static conditions. However, it should be noted that the mutants were less spiral than the wild-type and the reduced autoaggregation and biofilm forming ability could be a result of the changed morphology of the mutants.

*T. denticola* and *P. gingivalis* W50 form dual-species biofilms synergistically. *T. denticola* periplasmic flagella were shown to be important for synergistic biofilm formation with *P. gingivalis*. In agreement with previous studies (Vesey and Kuramitsu, 2004; Yamada et al., 2005), the absence of periplasmic flagella in Δ*flgE* attenuated its biofilm forming ability with *P. gingivalis* in static biofilm assays. Δ*flgE* also displayed a reduced rate of coaggregation with *P. gingivalis*. Although the abundance of the surface proteins involved in the binding of *T. denticola* and *P. gingivalis*, including dentilisin and the major sheath protein (Hashimoto et al., 2003; Rosen et al., 2008), in Δ*flgE* was comparable to those of wild-type, there could be changes in other unidentified surface components that are involved in the binding of *T. denticola* and *P. gingivalis*. Interestingly, the dual-species biofilms of Δ*flgE* grown together with *P. gingivalis* were smaller than the mono-species biofilms of *P. gingivalis*, suggesting an inhibitory effect from the absence of periplasmic flagella to dual-species biofilm formation under static conditions. This appears to be further supported by the observation that Δ*motB* contains a lower number of periplasmic flagella and reduced spirality compared with WT and produces a dual species biofilm intermediate in size compared with those of flagellated WT and the unflagellated Δ*flgE*. However, caution is required when drawing such conclusions about the effects of a reduced number of flagella in Δ*motB* dual species biofilm since the absence of MotB also had extensive effects on proteins not directly involved in motility, including changes in expression of several major surface proteins which may also effect dual species biofilm production.

The comprehensive quantitative analysis of the proteomes of *T. denticola* mutants revealed the wide effects of motility gene inactivation on *T. denticola* proteomes; this technique may be used to help identify changes in protein expression that contribute to mutant phenotypes. *T. denticola* periplasmic flagella were shown to be important for synergistic biofilm formation with *P. gingivalis*.

## Data Availability Statement

The datasets generated for this study can be found in the NCBI Bioproject accession number PRJNA561478.

## Author Contributions

HN conducted the majority of the benchwork as part of her Ph.D. under the supervision of NS, SD, and CB. SD, NS, CB, and ER devised the project. The mutant creation and confirmation were conducted by NS, CB, BH, and HN. Proteomic analyses of the mutants were carried out by PV. Y-YC carried out the electron microscopy imaging and analyses. SL and HN executed the biofilm analyses. HN, SD, CB, NS, and ER interpreted the results and wrote the manuscript.

### Conflict of Interest

The authors declare that the research was conducted in the absence of any commercial or financial relationships that could be construed as a potential conflict of interest.

## Supplementary Material

Supplementary Figure 1 A schematic representation of the mutational approach **(A)** *flgE* mutant construction **(B)** *motB* mutant construction.

Supplementary Table 1 Primers used in this study.

Supplementary Table 2 Nucleotide polymorphisms of *T. denticola* 33520 mutants determined by genomic sequencing.

Supplementary Table 3 Proteins significantly changed in abundance in *T. denticola* Δ*flgE* mutant relative to wild-type (ratio ≥1.5 and ≤0.67, *p* < 0.05).

Supplementary Table 4 Proteins significantly changed in abundance in *T. denticola* Δ*motB* mutant relative to wild-type (ratio ≥1.5 and ≤0.67, *p* < 0.05).

Supplementary Table 5 Proteins that were not detected in *T. denticola* ATCC 33520 but were detected in Δ*motB* (*p* < 0.05).
