# Supplementary figures and images for: The Role of Treponema denticola Motility in Synergistic Biofilm Formation With Porphyromonas gingivalis
Source: Front Cell Infect Microbiol. 2019 Dec 18;9:432. doi: 10.3389/fcimb.2019.00432 (PMC6930189; doi:10.3389/fcimb.2019.00432)

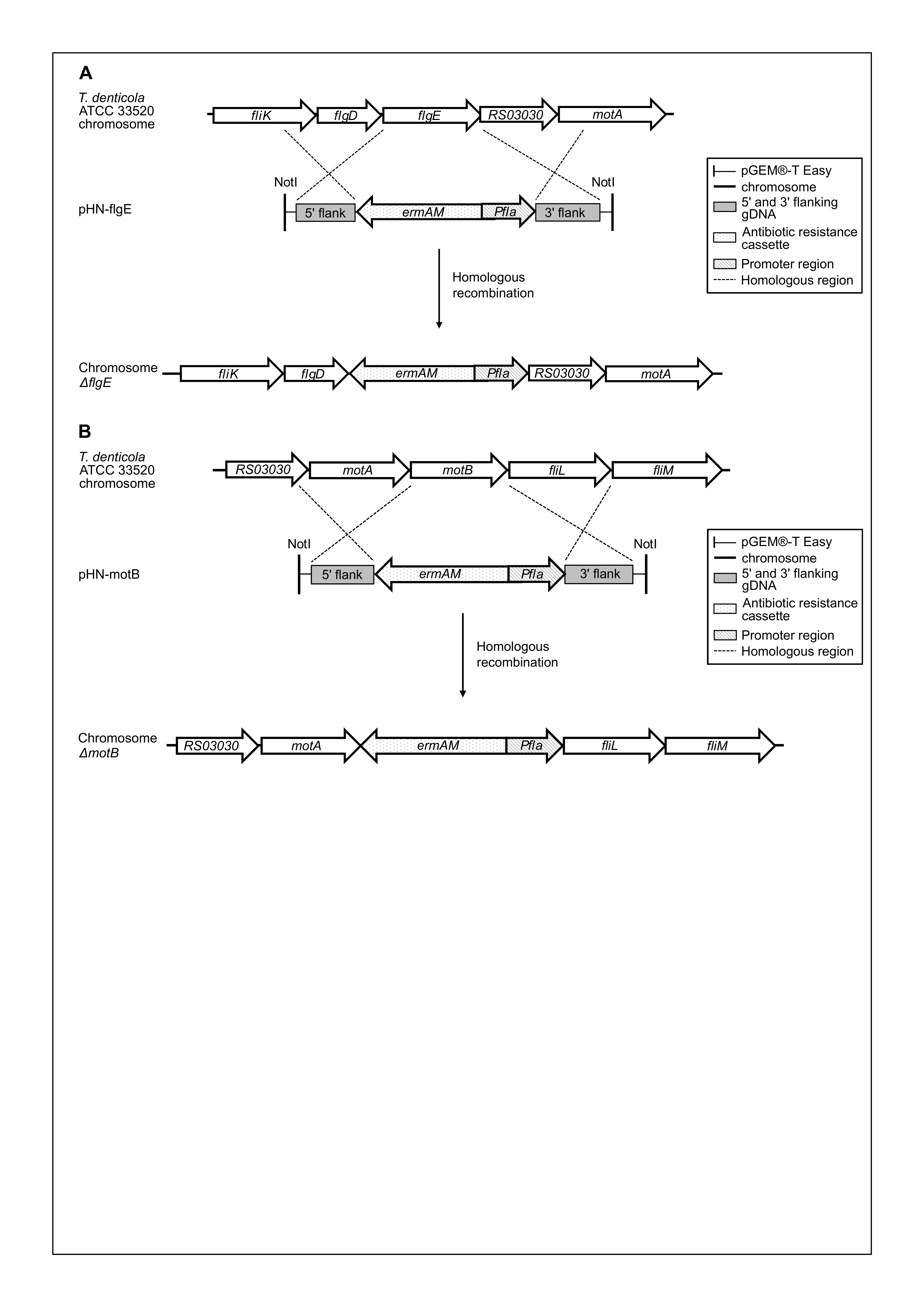

Supplement: Supplementary Figure 1 — A schematic representation of the mutational approach (A) flgE mutant construction (B) motB mutant construction. [file Image_1.TIF]
