# Supplementary material for: The Role of Treponema denticola Motility in Synergistic Biofilm Formation With Porphyromonas gingivalis
Source: Front Cell Infect Microbiol. 2019 Dec 18;9:432. doi: 10.3389/fcimb.2019.00432 (PMC6930189; doi:10.3389/fcimb.2019.00432)
Supplement: Supplementary Table 1 — Primers used in this study. [file Table_1.DOCX]

Supplementary Table 1. Primers used in this study.

| **Primer** | **Sequence (5’** 🡪 **3’)** | **Description** |
| --- | --- | --- |
| TDE2766-F | AGGAATATTCGGAGGTATAGCAG | Forward primer to amplify upstream region of *motB* |
| TDE2766-R | CCTCTAGAGTCGACCTGCAGCCTTGCCATTTTAATCCTTTAATAC | Reverse primer to amplify upstream region of *motB*, fused with downstream region of *ermAM* indicated by underline |
| ermAM-2766-F | AAAGGATTAAAATGGCAAGGCTGCAGGTCGACTCTAGAG | Forward primer to amplify *ermAM*, fused with upstream region of *motB* indicated by underline |
| fla prom-2764-R | ATTAAGTCATTATCAGCCATATGAACCTCCATAAAAACTTTTTGCAG | Reverse primer to amplify *fla* promoter, fused with downstream region of *motB* indicated by underline |
| TDE2764-F | AAGTTTTTATGGAGGTTCATATGGCTGATAATGACTTAATGGATG | Forward primer to amplify downstream region of *motB*, fused with *fla* promoter indicated by underline |
| TDE2764-R | TCTGTATCCGTATCTCCTGAG | Reverse primer to amplify downstream region of *motB* |
| TDE2768-F | TGTTGCCTCTAAAGAAGCCTAC | Forward primer to amplify upstream region of *flgE* |
| TDE2768-R | CCTCTAGAGTCGACCTGCAGTAATTATTGCCTCCTAATTGTTATCTG | Reverse primer to amplify upstream region of *flgE*, fused with downstream region of *ermAM* indicated by underline |
| ermAM-2768-F | CAATTAGGAGGCAATAATTACTGCAGGTCGACTCTAGAG | Forward primer to amplify *ermAM*, fused with upstream region of *flgE* indicated by underline |
| fla prom-2767-R | AGCCGCGTTACCTGTATCATATGAACCTCCATAAAAACTTTTTGCAG | Reverse primer to amplify *fla* promoter, fused with downstream region of *flgE* indicated by underline |
| TDE2767-F (flgE KO) | AAGTTTTTATGGAGGTTCATATGATACAGGTAACGCGGCTAAAC | Forward primer to amplify downstream region of *flgE*, fused with *fla* promoter indicated by underline |
| TDE2767-R (flgE KO) | AGCATTTACTAAGGATATCCATGTG | Reverse primer to amplify downstream region of *flgE* |
| 5’motB-F | TATTGAGTGTAATCCGGATGTTAC | Forward primer to validate *motB*-deleted mutant |
| 3’motB-R | TACGTAAGCTGTTCTACGGTTG | Reverse primer to validate *motB*-deleted mutant |
| 5’flgE-F | GATGCTTCAGCAGACTTTGTAC | Forward primer to validate *flgE*-deleted mutant |
| 3’flgE-R | CATGTTAGGGCCGAGAGCA | Reverse primer to validate *flgE*-deleted mutant |
