# Supplementary material for: The Role of Treponema denticola Motility in Synergistic Biofilm Formation With Porphyromonas gingivalis
Source: Front Cell Infect Microbiol. 2019 Dec 18;9:432. doi: 10.3389/fcimb.2019.00432 (PMC6930189; doi:10.3389/fcimb.2019.00432)
Supplement: Supplementary Table 3 — Proteins significantly changed in abundance in T. denticola ΔflgE mutant relative to wild-type (ratio ≥1.5 and ≤0.67, p < 0.05). [file Table_3.DOCX]

**Supplementary Table 3. Proteins significantly changed in abundance in *T. denticola* *∆flgE* mutant relative to wild-type (ratio≥1.5 and ≤0.67, *p*<0.05).** Proteins predicted to be organized in an operon were shaded.

| Locus Tag | Protein description | Wild-type abundance^A^ | *∆flgE* ratio^B^ | *p* value | COG^C^ | Increased (+)/ decreased (-) in abundance in *∆flgE* |
| --- | --- | --- | --- | --- | --- | --- |
| TDE0051 | alcohol dehydrogenase, iron-containing | 2.40E+07 | 1.88 | 0.02 | C | + |
| TDE0114 | iron-dependent transcriptional regulator | 3.46E+07 | 0.55 | 0.01 | K | - |
| TDE0119 | flagellar protein FliS (fliS) | 1.43E+06 | 2.71 | 0.04 | N | + |
| TDE0173 | FtsK/SpoIIIE family protein | 1.91E+06 | 1.62 | 0.05 | D | + |
| TDE0174 | nicotinate phosphoribosyltransferase, putative | 2.88E+07 | 1.53 | 0.02 | H | + |
| TDE0383 | hypothetical protein | 5.62E+06 | 0.00 | 0.00 | S | - |
| TDE0423 | hypothetical protein | 1.85E+07 | 0.53 | 0.02 | - | - |
| TDE0446 | fibronectin type III domain protein | 5.53E+06 | 1.74 | 0.02 | S | + |
| TDE0455 | kinase, GHMP family | 2.11E+06 | 2.10 | 0.01 | G | + |
| TDE0463 | purine nucleoside phosphorylase (deoD) | 5.33E+07 | 0.62 | 0.03 | F | - |
| TDE0501 | hypothetical protein | 9.16E+06 | 0.50 | 0.01 | S | - |
| TDE0533 | hypothetical protein | 7.67E+06 | 0.44 | 0.04 | S | - |
| TDE0602 | 3-oxoacyl-(acyl-carrier-protein) synthase III (fabH) | 5.14E+06 | 0.45 | 0.03 | I | - |
| TDE0664 | OmpA family protein | 2.11E+07 | 2.87 | 0.00 | M | + |
| TDE0689 | 5-methylthioribose kinase | 1.51E+06 | 0.00 | 0.00 | S | - |
| TDE0758 | iron compound ABC transporter, periplasmic iron compound-binding protein, putative | 6.83E+06 | 0.23 | 0.01 | P | - |
| TDE0777 | ribosomal protein L14 (rplN) | 9.37E+07 | 0.66 | 0.01 | J | - |
| TDE0781 | ribosomal protein S8 (rpsH) | 4.62E+07 | 0.65 | 0.03 | J | - |
| TDE0823 | (3R)-hydroxymyristoyl-(acyl-carrier-protein) dehydratase, putative | 3.71E+07 | 0.56 | 0.03 | I | - |
| TDE0843 | conserved hypothetical protein | 1.88E+08 | 0.65 | 0.02 | S | - |
| TDE0845 | conserved hypothetical protein TIGR00266 | 6.15E+07 | 0.62 | 0.01 | S | - |
| TDE0984 | oligopeptide/dipeptide ABC transporter, permease protein, putative | 6.58E+06 | 0.00 | 0.00 | P | - |
| TDE0985 | oligopeptide/dipeptide ABC transporter, periplasmic peptide-binding protein, putative | 3.21E+08 | 0.33 | 0.00 | E | - |
| TDE0986 | oligopeptide/dipeptide ABC transporter, ATP-binding protein | 3.99E+06 | 0.00 | 0.02 | P | - |
| TDE1004 | flagellar filament core protein | 4.67E+08 | 0.01 | 0.00 | N | - |
| TDE1208 | DNA topoisomerase I (topA) | 6.93E+07 | 1.79 | 0.00 | L | + |
| TDE1211 | heat shock protein HslVU, ATPase subunit HslU (hslU) | 3.99E+07 | 2.02 | 0.00 | O | + |
| TDE1234 | hypothetical protein | 2.84E+05 | 1.51 | 0.02 | S | + |
| TDE1318 | hypothetical protein | 7.25E+05 | 0.00 | 0.01 | U | - |
| TDE1408 | flagellar filament outer layer protein FlaA, putative | 5.77E+08 | 0.04 | 0.00 | N | - |
| TDE1409 | flagellar filament outer layer protein FlaA, putative | 5.59E+08 | 0.04 | 0.00 | N | - |
| TDE1475 | flagellar filament core protein | 4.47E+07 | 0.00 | 0.00 | N | - |
| TDE1483 | conserved hypothetical protein | 1.28E+08 | 0.30 | 0.00 | S | - |
| TDE1521 | hydro-lyase, tartrate/fumarate family, beta subunit | 5.29E+07 | 0.60 | 0.02 | C | - |
| TDE1640 | 3-dehydroquinate dehydratase, type 1, putative/shikimate 5-dehydrogenase, putative | 5.43E+06 | 1.59 | 0.03 | E | + |
| TDE1684 | adenine phosphoribosyltransferase | 7.01E+06 | 0.67 | 0.03 | F | - |
| TDE1712 | flagellar filament outer layer protein (flaA) | 1.85E+09 | 0.02 | 0.00 | N | - |
| TDE1727 | conserved hypothetical protein | 1.03E+08 | 0.46 | 0.00 | O | - |
| TDE1754 | desulfoferrodoxin/neelaredoxin | 7.38E+07 | 0.54 | 0.04 | C | - |
| TDE1884 | hypothetical protein | 2.66E+06 | 2.37 | 0.00 | S | + |
| TDE1916 | glycerol kinase (glpK) | 1.03E+07 | 1.85 | 0.02 | C | + |
| TDE1917 | cytidylyltransferase domain protein | 2.72E+06 | 1.88 | 0.00 | M | + |
| TDE1918 | conserved hypothetical protein | 2.05E+06 | 1.58 | 0.02 | S | + |
| TDE1919 | conserved domain protein | 1.64E+07 | 1.90 | 0.01 | S | + |
| TDE2030 | lipoprotein, RlpA family | 5.45E+07 | 1.58 | 0.04 | M | + |
| TDE2043 | signal recognition particle-docking protein FtsY (ftsY) | 5.55E+06 | 0.64 | 0.04 | U | - |
| TDE2048 | conserved hypothetical protein | 1.78E+07 | 1.70 | 0.01 | S | + |
| TDE2085 | amino acid kinase family protein | 7.68E+07 | 1.55 | 0.02 | F | + |
| TDE2087 | translation initiation factor IF-1 (infA) | 4.30E+07 | 0.61 | 0.05 | J | - |
| TDE2130 | hypothetical protein | 2.18E+07 | 0.00 | 0.03 | - | - |
| TDE2198 | pyruvate-ferredoxin oxidoreductase | 2.85E+07 | 1.59 | 0.00 | C | + |
| TDE2302 | HD domain protein | 1.09E+07 | 0.64 | 0.02 | T | - |
| TDE2353 | flagellar hook-associated protein 3 | 1.83E+06 | 0.59 | 0.00 | N | - |
| TDE2565 | hypothetical protein | 2.25E+06 | 0.00 | 0.00 | S | - |
| TDE2611 | conserved hypothetical protein | 2.62E+06 | 1.52 | 0.03 | S | + |
| TDE2644 | pyridine nucleotide-disulphide oxidoreductase family protein | 4.17E+05 | 0.00 | 0.01 | O | - |
| TDE2693 | ankyrin repeat protein | 3.86E+07 | 2.06 | 0.00 | S | + |
| TDE2721 | helicase domain protein | 3.38E+06 | 0.67 | 0.01 | L | - |
| TDE2763 | flagellar motor switch protein FliM (fliM) | 1.47E+07 | 0.46 | 0.01 | N | - |
| TDE2764 | flagellar protein FliL (fliL) | 6.51E+07 | 0.50 | 0.00 | N | - |
| TDE2765 | flagellar motor rotation protein B (motB) | 3.44E+07 | 0.35 | 0.00 | N | - |
| TDE2766 | motility protein A (motA) | 3.46E+07 | 0.44 | 0.01 | N | - |
| TDE2768 | flagellar hook protein FlgE (flgE) | 4.39E+07 | 0.00 | 0.00 | N | - |
| TDE2779 | hypothetical protein | 5.98E+07 | 0.31 | 0.00 | S | - |

^A^ The abundance of each protein in the wild-type *T. denticola* ATCC 33520 was calculated from the average IBAQ intensity from three replicates.

^B^ Geometric mean of ratios, from three replicates, produced from the LFQ intensity of protein in *∆flgE* relative to that of protein in wild-type. Ratio of ≥1.5 indicates that the protein had increased in abundance in *∆flgE* relative to wild-type and ratio of ≤0.67 indicates that the protein had decreased in abundance in *∆flgE* relative to wild-type. Zero ratio indicates that the protein was identified in ATCC 33520 but not in *∆flgE*.

^C^ One-letter abbreviations for the functional COG categories: J, translation, ribosomal structure and biogenesis; K, transcription; L, replication, recombination and repair; D, cell cycle control, cell division, chromosome partitioning; V, defense mechanisms; T, signal transduction mechanisms; M, cell wall/membrane/envelope biogenesis; N, cell motility; U, intracellular trafficking, secretion, and vesicular transport; O, posttranslational modification, protein turnover, chaperones; C, energy production and conversion; G, carbohydrate transport and metabolism; E, amino acid transport and metabolism; F, nucleotide transport and metabolism; H, coenzyme transport and metabolism; I, lipid transport and metabolism; P, inorganic ion transport and metabolism; Q, secondary metabolites biosynthesis, transport and catabolism; R, general function prediction only; S, function unknown.
