# Supplementary material for: The Role of Treponema denticola Motility in Synergistic Biofilm Formation With Porphyromonas gingivalis
Source: Front Cell Infect Microbiol. 2019 Dec 18;9:432. doi: 10.3389/fcimb.2019.00432 (PMC6930189; doi:10.3389/fcimb.2019.00432)
Supplement: Supplementary Table 4 — Proteins significantly changed in abundance in T. denticola ΔmotB mutant relative to wild-type (ratio ≥1.5 and ≤0.67, p < 0.05). [file Table_4.DOCX]

**Supplementary Table 4. Proteins significantly changed in abundance in *T. denticola* *∆motB* mutant relative to wild-type (ratio≥1.5 and ≤0.67, *p*<0.05).** Proteins predicted to be organized in an operon were shaded. In the case where two or more operons were arranged consecutively, different darkness of shadings was used to differentiate the operons.

| Locus Tag | Protein description | Wild-type abundance^A^ | *∆motB* ratio^B^ | *p* value | COG^C^ | Increased (+)/ decreased (-) in abundance in *∆motB* |
| --- | --- | --- | --- | --- | --- | --- |
| TDE0008 | copper-translocating P-type ATPase | 1.74E+06 | 0.00 | 0.01 | P | - |
| TDE0011 | alkyl hydroperoxide reductase/peroxiredoxin | 5.73E+09 | 0.00 | 0.00 | O | - |
| TDE0012 | carbon starvation protein CstA, putative | 4.61E+08 | 0.01 | 0.00 | T | - |
| TDE0014 | conserved hypothetical protein | 7.18E+07 | 1.52 | 0.03 | S | + |
| TDE0034 | hypothetical protein | 1.57E+07 | 0.57 | 0.01 | - | - |
| TDE0042 | phosphate acetyltransferase (pta) | 2.71E+08 | 0.55 | 0.00 | C | - |
| TDE0043 | TPR domain protein | 3.53E+06 | 0.60 | 0.02 | U | - |
| TDE0047 | imidazolonepropionase (hutI) | 6.50E+07 | 0.61 | 0.01 | Q | - |
| TDE0048 | hypothetical protein | 1.85E+07 | 0.00 | 0.02 | - | - |
| TDE0055 | flagellar biosynthesis protein FlhA (flhA) | 2.86E+06 | 1.64 | 0.02 | U | + |
| TDE0064 | phosphofructokinase (pfk) | 4.43E+07 | 0.61 | 0.03 | G | - |
| TDE0065 | conserved hypothetical protein | 8.75E+06 | 0.00 | 0.03 | S | - |
| TDE0066 | conserved hypothetical protein | 6.33E+06 | 0.30 | 0.01 | S | - |
| TDE0072 | methyl-accepting chemotaxis protein | 3.62E+06 | 0.55 | 0.01 | T | - |
| TDE0081 | hypothetical protein | 1.74E+06 | 0.00 | 0.01 | S | - |
| TDE0089 | transglutaminase-like domain protein | 2.47E+07 | 0.48 | 0.03 | E | - |
| TDE0103 | aminotransferase, class-V | 1.79E+06 | 0.00 | 0.00 | E | - |
| TDE0114 | iron-dependent transcriptional regulator | 3.46E+07 | 0.59 | 0.00 | K | - |
| TDE0119 | flagellar protein FliS (fliS) | 1.43E+06 | 0.00 | 0.01 | U | - |
| TDE0129 | PyrBI protein (pyrBI) | 1.64E+08 | 2.23 | 0.01 | F | + |
| TDE0143 | thiamine ABC transporter, thiamine-binding protein | 5.36E+06 | 0.67 | 0.02 | H | - |
| TDE0149 | DNA-binding response regulator | 1.91E+06 | 0.00 | 0.00 | T | - |
| TDE0151 | integral membrane protein, YeeE/YedE family | 3.41E+07 | 0.47 | 0.02 | S | - |
| TDE0154 | adenylate/guanylate cyclase catalytic domain protein | 2.17E+06 | 0.00 | 0.00 | T | - |
| TDE0175 | pyrrolidone-carboxylate peptidase (pcp) | 9.88E+07 | 0.40 | 0.00 | O | - |
| TDE0183 | ABC transporter, permease protein | 6.05E+06 | 1.80 | 0.01 | P | + |
| TDE0186 | hypothetical protein | 5.97E+08 | 0.63 | 0.02 | P | - |
| TDE0200 | tetrapyrrole methylase family protein | 2.48E+06 | 0.00 | 0.01 | R | - |
| TDE0207 | permease, GntP family | 3.86E+07 | 0.38 | 0.00 | E | - |
| TDE0210 | cob(I)alamin adenosyltransferase (cobO) | 5.60E+07 | 1.56 | 0.04 | H | + |
| TDE0212 | conserved hypothetical protein | 1.23E+08 | 0.44 | 0.02 | P | - |
| TDE0213 | TPR domain protein | 3.61E+06 | 0.59 | 0.00 | U | - |
| TDE0239 | glycine reductase complex protein GrdD (grdD) | 2.31E+08 | 0.59 | 0.02 | I | - |
| TDE0294 | TrkA domain protein | 6.57E+06 | 0.54 | 0.04 | P | - |
| TDE0300 | cytosol aminopeptidase family protein | 3.88E+08 | 0.61 | 0.02 | E | - |
| TDE0301 | tetracenomycin polyketide synthesis O-methyltransferase TcmP, putative | 5.13E+06 | 0.00 | 0.02 | Q | - |
| TDE0336 | membrane protein, putative | 3.61E+06 | 1.52 | 0.04 | S | + |
| TDE0337 | glucosamine-6-phosphate isomerase (nagB) | 4.95E+07 | 0.41 | 0.01 | G | - |
| TDE0352 | ribose 5-phosphate isomerase B (rpiB) | 6.89E+07 | 0.35 | 0.00 | G | - |
| TDE0354 | general stress protein 14 | 1.82E+07 | 0.40 | 0.02 | R | - |
| TDE0358 | cinnamoyl ester hydrolase (cinI) | 2.59E+06 | 0.00 | 0.00 | I | - |
| TDE0374 | glycosyl transferase, group 1 family protein | 1.15E+06 | 0.00 | 0.00 | M | - |
| TDE0383 | hypothetical protein | 5.62E+06 | 0.00 | 0.00 | S | - |
| TDE0385 | ABC transporter, ATP-binding/permease protein | 0.00E+00 | NA^D^ | 0.02 | V | + |
| TDE0386 | ABC transporter, periplasmic substrate-binding protein | 5.86E+08 | 0.45 | 0.00 | P | - |
| TDE0389 | (R)-2-hydroxyglutaryl-CoA dehydratase, beta subunit, putative | 8.95E+07 | 0.48 | 0.01 | E | - |
| TDE0392 | (R)-2-hydroxyglutaryl-CoA dehydratase, beta subunit, putative | 3.44E+07 | 0.62 | 0.05 | E | - |
| TDE0398 | oligopeptide/dipeptide ABC transporter, periplasmic peptide-binding protein | 1.74E+07 | 0.47 | 0.01 | E | - |
| TDE0405 | major outer sheath protein | 3.40E+09 | 0.54 | 0.03 | M | - |
| TDE0418 | lipoprotein, putative | 3.02E+07 | 0.30 | 0.00 | - | - |
| TDE0419 | hypothetical protein | 2.72E+07 | 0.14 | 0.00 | - | - |
| TDE0423 | hypothetical protein | 1.85E+07 | 0.27 | 0.00 | - | - |
| TDE0425 | bacteriocin ABC transporter, ATP-binding/permease, putative | 7.31E+05 | 0.00 | 0.00 | V | - |
| TDE0448 | deoxyribose-phosphate aldolase (deoC) | 4.02E+07 | 0.55 | 0.03 | F | - |
| TDE0451 | arginine deiminase (arcA) | 9.24E+07 | 0.62 | 0.01 | E | - |
| TDE0463 | purine nucleoside phosphorylase (deoD) | 5.33E+07 | 0.55 | 0.00 | F | - |
| TDE0473 | heavy metal translocating P-type ATPase | 1.10E+06 | 0.00 | 0.01 | P | - |
| TDE0501 | hypothetical protein | 9.16E+06 | 0.00 | 0.00 | S | - |
| TDE0523 | conserved hypothetical protein | 4.61E+06 | 0.00 | 0.00 | S | - |
| TDE0536 | hypothetical protein | 3.20E+06 | 0.00 | 0.01 | S | - |
| TDE0540 | hypothetical protein | 1.22E+07 | 0.65 | 0.04 | S | - |
| TDE0544 | conserved hypothetical protein | 1.27E+07 | 0.41 | 0.00 | S | - |
| TDE0557 | hypothetical protein | 3.58E+06 | 0.00 | 0.00 | S | - |
| TDE0558 | hypothetical protein | 1.05E+07 | 0.00 | 0.00 | S | - |
| TDE0593 | internalin-related protein | 5.36E+05 | 0.00 | 0.00 | S | - |
| TDE0594 | cysteine protease domain, YopT-type | 2.56E+06 | 0.00 | 0.02 | - | - |
| TDE0601 | malonyl CoA-acyl carrier protein transacylase, putative | 2.38E+07 | 0.63 | 0.03 | I | - |
| TDE0605 | hypothetical protein | 7.28E+07 | 0.48 | 0.01 | - | - |
| TDE0607 | ParA family ATPase | 0.00E+00 | NA^D^ | 0.00 | D | + |
| TDE0615 | cobalamin biosynthesis protein CbiG (cbiG) | 1.61E+06 | 0.00 | 0.01 | H | - |
| TDE0626 | hypothetical protein | 5.23E+07 | 1.65 | 0.02 | S | + |
| TDE0630 | sigma factor regulatory protein | 6.25E+06 | 0.67 | 0.04 | T | - |
| TDE0631 | sigma factor regulatory protein, putative | 7.13E+06 | 0.41 | 0.01 | T | - |
| TDE0641 | UDP-N-acetylglucosamine 1-carboxyvinyltransferase (murA) | 2.79E+07 | 0.46 | 0.00 | M | - |
| TDE0649 | hypothetical protein | 1.69E+07 | 1.67 | 0.00 | - | + |
| TDE0650 | membrane protein, putative | 0.00E+00 | NA^D^ | 0.03 | R | + |
| TDE0651 | inorganic pyrophosphatase, frameshift mutation | 9.63E+07 | 1.82 | 0.01 | - | + |
| TDE0652 | membrane protein, putative | 4.04E+07 | 1.99 | 0.01 | S | + |
| TDE0654 | peptidase, M20/M25/M40 family | 1.68E+08 | 0.63 | 0.00 | R | - |
| TDE0665 | pyruvate ferredoxin/flavodoxin oxidoreductase family protein | 3.49E+08 | 1.92 | 0.00 | C | + |
| TDE0677 | conserved hypothetical protein | 1.26E+08 | 0.41 | 0.00 | S | - |
| TDE0689 | 5-methylthioribose kinase | 1.51E+06 | 0.00 | 0.01 | S | - |
| TDE0728 | FAD-binding protein, putative | 2.32E+06 | 0.00 | 0.01 | C | - |
| TDE0745 | glycine reductase complex selenoprotein GrdA | 2.75E+07 | 4.59 | 0.01 | C | + |
| TDE0758 | iron compound ABC transporter, periplasmic iron compound-binding protein, putative | 6.83E+06 | 4.14 | 0.01 | P | + |
| TDE0778 | ribosomal protein L24 (rplX) | 2.25E+06 | 1.82 | 0.00 | J | + |
| TDE0781 | ribosomal protein S8 (rpsH) | 4.62E+07 | 1.61 | 0.04 | J | + |
| TDE0782 | ribosomal protein L6 (rplF) | 1.63E+07 | 1.54 | 0.02 | J | + |
| TDE0783 | ribosomal protein L18 (rplR) | 3.95E+07 | 2.55 | 0.01 | J | + |
| TDE0784 | ribosomal protein S5 (rpsE) | 1.09E+08 | 0.65 | 0.04 | J | - |
| TDE0793 | conserved hypothetical protein | 2.25E+07 | 0.42 | 0.03 | S | - |
| TDE0799 | glycerophosphoryl diester phosphodiesterase, putative | 6.03E+06 | 0.49 | 0.03 | C | - |
| TDE0813 | ABC transporter, ATP-binding protein/permease | 1.06E+06 | 0.00 | 0.03 | V | - |
| TDE0834 | Na^+^-translocating NADH/quinone reductase, E subunit (nqrE) | 0.00E+00 | NA^D^ | 0.00 | C | + |
| TDE0840 | hypothetical protein | 7.92E+06 | 1.70 | 0.00 | S | + |
| TDE0853 | ribosomal protein S9 (rpsI) | 6.86E+07 | 2.36 | 0.02 | J | + |
| TDE0856 | L-fuculose phosphate aldolase, putative | 5.55E+06 | 2.14 | 0.01 | G | + |
| TDE0865 | conserved hypothetical protein | 8.13E+06 | 0.00 | 0.00 | S | - |
| TDE0870 | phosphatase/nucleotidase | 1.15E+08 | 0.64 | 0.03 | F | - |
| TDE0872 | recA protein (recA) | 6.32E+06 | 2.12 | 0.01 | L | + |
| TDE0877 | conserved hypothetical protein | 1.52E+07 | 0.46 | 0.02 | I | - |
| TDE0880 | nicotinate-nucleotide-dimethylbenzimidazole phosphoribosyltransferase (cobT) | 1.50E+06 | 0.00 | 0.03 | H | - |
| TDE0885 | ribosomal protein L19 (rplS) | 1.18E+08 | 1.69 | 0.05 | J | + |
| TDE0951 | lipoprotein, putative | 9.19E+07 | 0.63 | 0.02 | R | - |
| TDE0957 | glycerophosphoryl diester phosphodiesterase family protein | 6.84E+06 | 0.00 | 0.01 | C | - |
| TDE0971 | arginyl-tRNA synthetase (argS) | 1.67E+07 | 0.67 | 0.01 | J | - |
| TDE0983 | oligopeptide/dipeptide ABC permease, frameshift mutation | 0.00E+00 | NA^D^ | 0.00 | - | + |
| TDE0984 | oligopeptide/dipeptide ABC transporter, permease protein, putative | 6.58E+06 | 8.79 | 0.01 | R | + |
| TDE0985 | oligopeptide/dipeptide ABC transporter, periplasmic peptide-binding protein, putative | 3.21E+08 | 5.20 | 0.00 | E | + |
| TDE0986 | oligopeptide/dipeptide ABC transporter, ATP-binding protein | 3.99E+06 | 12.92 | 0.00 | P | + |
| TDE0987 | oligopeptide/dipeptide ABC transporter, ATP-binding protein | 0.00E+00 | NA^D^ | 0.00 | E | + |
| TDE1001 | orotate phosphoribosyltransferase (pyrE) | 2.79E+07 | 0.61 | 0.04 | F | - |
| TDE1004 | flagellar filament core protein | 4.67E+08 | 0.24 | 0.00 | N | - |
| TDE1009 | methyl-accepting chemotaxis protein | 2.84E+07 | 1.68 | 0.01 | T | + |
| TDE1016 | probable ATP-dependent protease LA, frameshift mutation | 3.19E+06 | 0.52 | 0.02 | - | - |
| TDE1020 | dicarboxylate transporter, periplasmic dicarboxylate-binding protein, putative | 5.99E+07 | 0.59 | 0.02 | G | - |
| TDE1021 | lipoprotein, putative | 1.45E+07 | 0.00 | 0.00 | S | - |
| TDE1027 | ribonuclease III (rnc) | 1.78E+06 | 0.00 | 0.00 | K | - |
| TDE1049 | translation elongation factor G (fusA-2) | 1.27E+08 | 1.50 | 0.00 | J | + |
| TDE1072 | lipoprotein, putative | 6.89E+08 | 0.60 | 0.02 | R | - |
| TDE1075 | oligopeptide/dipeptide ABC transporter, ATP-binding protein | 2.61E+07 | 0.43 | 0.01 | P | - |
| TDE1076 | oligopeptide/dipeptide ABC transporter, ATP-binding protein | 4.65E+07 | 0.55 | 0.00 | E | - |
| TDE1086 | conserved hypothetical protein TIGR00255 | 7.88E+06 | 0.56 | 0.02 | S | - |
| TDE1088 | conserved hypothetical protein | 6.96E+06 | 0.00 | 0.00 | S | - |
| TDE1089 | conserved hypothetical protein | 3.99E+07 | 0.48 | 0.02 | S | - |
| TDE1094 | conserved hypothetical protein | 1.49E+06 | 0.00 | 0.00 | G | - |
| TDE1102 | hypothetical protein | 8.91E+07 | 0.59 | 0.00 | S | - |
| TDE1111 | transporter, putative | 5.87E+06 | 0.00 | 0.00 | C | - |
| TDE1122 | anti-anti-sigma factor | 1.80E+07 | 0.00 | 0.01 | T | - |
| TDE1124 | hypothetical protein | 1.01E+07 | 1.85 | 0.03 | S | + |
| TDE1174 | hypothetical protein | 2.09E+07 | 0.49 | 0.00 | S | - |
| TDE1197 | conserved hypothetical protein TIGR00242 | 5.09E+06 | 1.91 | 0.02 | S | + |
| TDE1198 | S-adenosyl-methyltransferase MraW (mraW) | 8.30E+06 | 1.77 | 0.03 | M | + |
| TDE1206 | TPR domain protein | 1.94E+07 | 2.14 | 0.00 | S | + |
| TDE1208 | DNA topoisomerase I (topA) | 6.93E+07 | 1.93 | 0.00 | L | + |
| TDE1226 | zinc ABC transporter, periplasmic zinc-binding protein (troA) | 1.22E+07 | 0.58 | 0.02 | P | - |
| TDE1231 | hypothetical protein | 3.27E+07 | 0.61 | 0.02 | S | - |
| TDE1234 | hypothetical protein | 2.84E+05 | 3.81 | 0.04 | S | + |
| TDE1236 | triosephosphate isomerase (tpiA) | 6.67E+07 | 0.60 | 0.01 | G | - |
| TDE1259 | amino acid carrier family protein | 1.52E+08 | 1.81 | 0.02 | E | + |
| TDE1271 | oligopeptide/dipeptide ABC transporter, ATP-binding protein | 7.54E+05 | 0.00 | 0.00 | E | - |
| TDE1273 | oligopeptide/dipeptide ABC transporter, peptide-binding protein | 3.20E+08 | 0.62 | 0.01 | E | - |
| TDE1277 | Fe-hydrogenase large subunit family protein | 0.00E+00 | NA^D^ | 0.01 | C | + |
| TDE1299 | conserved hypothetical protein | 8.09E+06 | 0.64 | 0.03 | S | - |
| TDE1300 | conserved hypothetical protein | 2.06E+07 | 0.41 | 0.03 | S | - |
| TDE1314 | penicillin-binding protein | 8.47E+05 | 0.00 | 0.04 | M | - |
| TDE1318 | hypothetical protein | 7.25E+05 | 0.00 | 0.01 | U | - |
| TDE1328 | hypothetical protein | 9.56E+06 | 0.00 | 0.00 | S | - |
| TDE1333 | hflC protein, putative | 3.11E+07 | 0.67 | 0.02 | O | - |
| TDE1342 | conserved hypothetical protein | 2.16E+06 | 0.00 | 0.01 | S | - |
| TDE1349 | rod shape-determining protein MreB (mreB) | 3.36E+06 | 2.78 | 0.00 | D | + |
| TDE1352 | penicillin-binding protein | 6.63E+05 | 0.00 | 0.02 | M | - |
| TDE1364 | valyl-tRNA synthetase (valS) | 1.26E+07 | 1.51 | 0.04 | J | + |
| TDE1370 | YjeF-related protein | 1.34E+06 | 0.00 | 0.01 | G | - |
| TDE1373 | HD domain protein | 1.70E+06 | 1.93 | 0.04 | S | + |
| TDE1388 | conserved hypothetical protein | 0.00E+00 | NA^D^ | 0.00 | S | + |
| TDE1398 | conserved hypothetical protein | 9.77E+06 | 0.53 | 0.01 | L | - |
| TDE1399 | prolipoprotein diacylglyceryl transferase (lgt) | 2.29E+06 | 0.00 | 0.05 | M | - |
| TDE1407 | conserved hypothetical protein | 9.91E+05 | 0.00 | 0.01 | S | - |
| TDE1408 | flagellar filament outer layer protein FlaA, putative | 5.77E+08 | 0.29 | 0.00 | N | - |
| TDE1409 | flagellar filament outer layer protein FlaA, putative | 5.59E+08 | 0.28 | 0.00 | N | - |
| TDE1415 | nucleotidyl transferase/aminotransferase, class V | 3.26E+07 | 1.57 | 0.00 | E | + |
| TDE1416 | ABC transporter, permease protein | 2.63E+06 | 0.00 | 0.02 | M | - |
| TDE1432 | conserved domain protein | 0.00E+00 | NA^D^ | 0.00 | S | + |
| TDE1435 | hypothetical protein | 1.23E+06 | 0.00 | 0.00 | S | - |
| TDE1444 | metallo-beta-lactamase family protein | 1.60E+07 | 2.59 | 0.02 | J | + |
| TDE1460 | conserved domain protein | 1.64E+07 | 0.00 | 0.00 | S | - |
| TDE1464 | SsrA-binding protein (smpB) | 7.13E+06 | 0.00 | 0.00 | O | - |
| TDE1467 | HD domain protein | 1.11E+06 | 1.94 | 0.04 | T | + |
| TDE1468 | glycoprotease family protein | 3.42E+06 | 0.00 | 0.01 | O | - |
| TDE1477 | flagellar filament core protein | 8.93E+08 | 0.27 | 0.00 | N | - |
| TDE1478 | conserved hypothetical protein | 8.75E+06 | 0.00 | 0.01 | S | - |
| TDE1480 | conserved hypothetical protein | 1.89E+08 | 0.07 | 0.02 | S | - |
| TDE1483 | conserved hypothetical protein | 1.28E+08 | 1.52 | 0.02 | S | + |
| TDE1484 | hypothetical protein | 6.30E+06 | 0.00 | 0.01 | - | - |
| TDE1489 | hypothetical protein | 3.21E+07 | 0.64 | 0.04 | - | - |
| TDE1490 | hypothetical protein | 2.72E+06 | 1.59 | 0.05 | S | + |
| TDE1493 | chemotaxis protein CheX (cheX) | 1.78E+08 | 0.41 | 0.01 | N | - |
| TDE1494 | chemotaxis protein CheY (cheY) | 1.71E+08 | 0.53 | 0.03 | T | - |
| TDE1496 | chromosome partition protein SmC, putative | 5.18E+06 | 2.01 | 0.01 | D | + |
| TDE1499 | adenylosuccinate lyase, putative | 5.29E+07 | 1.66 | 0.03 | F | + |
| TDE1503 | transcription termination factor Rho (rho) | 3.24E+07 | 1.63 | 0.01 | K | + |
| TDE1509 | lipoprotein releasing system, ATP-binding protein, putative | 1.46E+06 | 0.00 | 0.01 | V | - |
| TDE1511 | pathogen-specific surface antigen, putative | 1.55E+09 | 0.54 | 0.00 | P | - |
| TDE1519 | conserved hypothetical protein | 5.95E+07 | 0.51 | 0.00 | S | - |
| TDE1533 | conserved hypothetical protein | 2.62E+06 | 0.00 | 0.01 | S | - |
| TDE1541 | metallo-beta-lactamase family protein | 1.29E+07 | 2.25 | 0.01 | S | + |
| TDE1548 | conserved hypothetical protein TIGR00103 | 5.31E+06 | 0.00 | 0.00 | S | - |
| TDE1584 | lipoprotein, putative | 3.84E+08 | 2.30 | 0.01 | S | + |
| TDE1588 | tryptophanyl-tRNA synthetase, putative | 0.00E+00 | NA^D^ | 0.00 | J | + |
| TDE1616 | glycerate dehydrogenase (hprA) | 1.13E+07 | 1.83 | 0.01 | C | + |
| TDE1628 | hypothetical protein | 5.90E+07 | 2.91 | 0.00 | - | + |
| TDE1639 | sporulation related repeat protein | 5.86E+07 | 0.56 | 0.01 | S | - |
| TDE1643 | PASTA domain protein | 9.70E+06 | 1.50 | 0.03 | T | + |
| TDE1657 | N utilization substance protein B (nusB) | 8.37E+06 | 0.00 | 0.00 | K | - |
| TDE1668 | amino acid permease family protein | 2.25E+08 | 0.00 | 0.01 | E | - |
| TDE1669 | hemolysin | 1.26E+09 | 0.00 | 0.00 | E | - |
| TDE1671 | trigger factor (tig) | 9.09E+07 | 1.58 | 0.03 | O | + |
| TDE1672 | ATP-dependent Clp protease, proteolytic subunit ClpP (clpP-1) | 1.17E+07 | 2.24 | 0.00 | U | + |
| TDE1678 | ribosomal protein S6 (rpsF) | 6.64E+07 | 1.85 | 0.01 | J | + |
| TDE1687 | conserved hypothetical protein | 8.83E+06 | 1.84 | 0.01 | S | + |
| TDE1699 | hypothetical protein | 3.12E+06 | 0.00 | 0.01 | - | - |
| TDE1712 | flagellar filament outer layer protein (flaA) | 1.85E+09 | 0.18 | 0.01 | N | - |
| TDE1713 | hypothetical protein | 0.00E+00 | NA^D^ | 0.00 | S | + |
| TDE1725 | conserved domain protein | 1.37E+06 | 0.00 | 0.01 | S | - |
| TDE1726 | polyA polymerase family protein | 4.51E+06 | 2.23 | 0.04 | J | + |
| TDE1727 | conserved hypothetical protein | 1.03E+08 | 0.54 | 0.04 | O | - |
| TDE1730 | glycosyl hydrolase, family 2 | 0.00E+00 | NA^D^ | 0.00 | G | + |
| TDE1745 | conserved hypothetical protein | 6.72E+06 | 0.00 | 0.01 | S | - |
| TDE1748 | GTP-binding protein, GTP1/Obg family | 1.59E+06 | 0.00 | 0.00 | S | - |
| TDE1754 | desulfoferrodoxin/neelaredoxin | 7.38E+07 | 2.71 | 0.01 | C | + |
| TDE1848 | hypothetical protein | 1.83E+07 | 0.57 | 0.04 | S | - |
| TDE1849 | hypothetical protein | 4.56E+07 | 0.31 | 0.01 | S | - |
| TDE1858 | transcriptional regulator, PadR family | 1.22E+07 | 0.00 | 0.01 | K | - |
| TDE1859 | hypothetical protein | 4.51E+07 | 0.37 | 0.00 | S | - |
| TDE1862 | conserved domain protein | 2.45E+08 | 0.52 | 0.02 | S | - |
| TDE1881 | methyltransferase, putative | 2.94E+06 | 0.00 | 0.01 | L | - |
| TDE1882 | glycosyl hydrolase, family 1 | 1.54E+07 | 1.81 | 0.05 | G | + |
| TDE1896 | phosphoribosylaminoimidazolecarboxamide formyltransferase/IMP cyclohydrolase (purH) | 8.42E+06 | 1.56 | 0.01 | F | + |
| TDE1898 | preprotein translocase, SecA subunit (secA) | 2.85E+07 | 1.55 | 0.00 | U | + |
| TDE1914 | Holliday junction DNA helicase RuvB (ruvB) | 1.73E+06 | 0.00 | 0.01 | L | - |
| TDE1919 | conserved domain protein | 1.64E+07 | 0.63 | 0.03 | S | - |
| TDE1927 | phenylalanyl-tRNA synthetase, beta subunit (pheT) | 1.63E+07 | 1.71 | 0.04 | J | + |
| TDE1969 | sigma-54 dependent transcriptional regulator/response regulator | 1.96E+06 | 0.50 | 0.01 | T | - |
| TDE1986 | adenylate cyclase, putative | 2.64E+07 | 0.57 | 0.02 | T | - |
| TDE2009 | conserved hypothetical protein | 4.33E+06 | 0.00 | 0.02 | S | - |
| TDE2029 | hydrolase, TatD family | 2.70E+06 | 0.00 | 0.00 | L | - |
| TDE2039 | lipoprotein releasing system, permease protein, putative | 2.80E+06 | 0.00 | 0.00 | M | - |
| TDE2043 | signal recognition particle-docking protein FtsY (ftsY) | 5.55E+06 | 0.00 | 0.02 | U | - |
| TDE2061 | conserved hypothetical protein | 1.13E+07 | 0.00 | 0.01 | P | - |
| TDE2073 | TPR domain protein | 5.34E+07 | 0.52 | 0.00 | G | - |
| TDE2074 | radical SAM enzyme, Cfr family | 2.66E+06 | 0.00 | 0.00 | R | - |
| TDE2079 | sigma-54 dependent transcriptional regulator, putative | 1.44E+06 | 0.00 | 0.01 | T | - |
| TDE2085 | amino acid kinase family protein | 7.68E+07 | 0.46 | 0.01 | F | - |
| TDE2087 | translation initiation factor IF-1 (infA) | 4.30E+07 | 1.68 | 0.04 | J | + |
| TDE2089 | signal recognition particle protein (ffh) | 8.52E+05 | 0.00 | 0.01 | U | - |
| TDE2092 | amino acid ABC transporter, amino acid-binding protein, putative | 1.35E+06 | 0.00 | 0.00 | T | - |
| TDE2093 | conserved hypothetical protein | 2.15E+07 | 0.41 | 0.04 | S | - |
| TDE2115 | HAMP domain protein | 6.67E+06 | 0.56 | 0.00 | T | - |
| TDE2120 | glycine reductase complex proprotein GrdE2 (grdE-2) | 4.60E+08 | 0.59 | 0.01 | S | - |
| TDE2144 | conserved hypothetical protein | 1.15E+07 | 0.31 | 0.02 | S | - |
| TDE2148 | OmpA family protein | 7.73E+06 | 0.34 | 0.01 | M | - |
| TDE2167 | pyridine nucleotide-disulphide oxidoreductase family protein | 1.01E+07 | 2.27 | 0.00 | E | + |
| TDE2183 | hypothetical protein | 2.53E+07 | 0.67 | 0.03 | S | - |
| TDE2189 | M23/M37 peptidase domain protein | 6.48E+06 | 0.47 | 0.01 | M | - |
| TDE2200 | methionine gamma-lyase (megL) | 5.96E+08 | 0.65 | 0.02 | E | - |
| TDE2210 | hypothetical protein | 3.56E+07 | 0.51 | 0.01 | S | - |
| TDE2217 | galactose/glucose-binding lipoprotein (mglB) | 2.54E+08 | 0.59 | 0.00 | G | - |
| TDE2226 | ABC transporter, substrate-binding protein, putative | 2.18E+07 | 0.48 | 0.04 | P | - |
| TDE2235 | methylaspartate ammonia-lyase | 1.23E+08 | 2.40 | 0.01 | E | + |
| TDE2244 | conserved hypothetical protein | 1.81E+06 | 3.38 | 0.02 | S | + |
| TDE2257 | 5-nucleotidase family protein | 8.45E+08 | 0.60 | 0.00 | F | - |
| TDE2280 | transglycosylase, SLT family | 5.22E+06 | 0.32 | 0.01 | M | - |
| TDE2281 | LysM domain protein | 5.03E+07 | 1.68 | 0.05 | S | + |
| TDE2285 | conserved hypothetical protein | 0.00E+00 | NA^D^ | 0.01 | S | + |
| TDE2302 | HD domain protein | 1.09E+07 | 0.00 | 0.04 | T | - |
| TDE2325 | conserved hypothetical protein | 0.00E+00 | NA^D^ | 0.00 | S | + |
| TDE2328 | efflux pump component MtrF (mtrF) | 2.39E+07 | 1.62 | 0.03 | H | + |
| TDE2342 | 1-deoxy-D-xylulose 5-phosphate reductoisomerase (dxr) | 1.29E+06 | 0.00 | 0.00 | I | - |
| TDE2344 | undecaprenyl diphosphate synthase (uppS) | 2.08E+06 | 0.00 | 0.00 | I | - |
| TDE2346 | translation elongation factor Ts (tsf) | 1.08E+08 | 0.60 | 0.00 | J | - |
| TDE2352 | flagellar hook-associated protein FlgK (flgK) | 2.08E+07 | 0.58 | 0.00 | N | - |
| TDE2353 | flagellar hook-associated protein 3 | 1.83E+06 | 0.00 | 0.01 | N | - |
| TDE2356 | iron compound ABC transporter, periplasmic iron compound-binding protein, putative | 5.15E+06 | 0.00 | 0.00 | P | - |
| TDE2357 | iron compound ABC transporter, permease protein | 4.94E+06 | 0.00 | 0.00 | P | - |
| TDE2376 | conserved hypothetical protein | 3.88E+06 | 0.00 | 0.00 | S | - |
| TDE2390 | hypothetical protein | 3.36E+08 | 0.39 | 0.01 | - | - |
| TDE2391 | peptidyl-prolyl cis-trans isomerase | 1.93E+08 | 0.63 | 0.03 | O | - |
| TDE2392 | hypothetical protein | 2.26E+07 | 0.66 | 0.04 | S | - |
| TDE2410 | hemolysin | 1.64E+08 | 0.12 | 0.01 | E | - |
| TDE2414 | hypothetical protein | 7.56E+06 | 0.00 | 0.00 | - | - |
| TDE2451 | S-adenosylmethionine:tRNA ribosyltransferase-isomerase (queA) | 9.49E+05 | 0.00 | 0.01 | J | - |
| TDE2466 | conserved hypothetical protein | 2.77E+07 | 0.00 | 0.00 | O | - |
| TDE2469 | hexokinase family protein | 2.27E+06 | 0.00 | 0.01 | G | - |
| TDE2480 | chaperone protein HtpG (htpG) | 8.02E+07 | 2.13 | 0.00 | O | + |
| TDE2485 | conserved hypothetical protein | 2.76E+07 | 0.65 | 0.04 | S | - |
| TDE2501 | response regulator | 1.64E+07 | 0.61 | 0.02 | T | - |
| TDE2503 | conserved domain protein | 2.09E+06 | 0.00 | 0.02 | S | - |
| TDE2504 | O-sialoglycoprotein endopeptidase (gcp) | 2.47E+06 | 0.00 | 0.00 | O | - |
| TDE2515; TDE0945 | conserved hypothetical protein;;conserved hypothetical protein | 2.49E+06 | 0.00 | 0.02 | S | - |
| TDE2517 | DNA repair protein RadA (radA) | 3.84E+06 | 0.43 | 0.00 | O | - |
| TDE2536 | conserved hypothetical protein | 1.13E+07 | 0.65 | 0.03 | S | - |
| TDE2542 | antigen, putative | 2.99E+06 | 0.00 | 0.01 | - | - |
| TDE2549 | methyl-accepting chemotaxis protein | 1.36E+06 | 7.13 | 0.01 | T | + |
| TDE2551 | hypothetical protein | 1.48E+06 | 0.00 | 0.01 | S | - |
| TDE2566 | conserved domain protein | 6.95E+06 | 1.73 | 0.01 | S | + |
| TDE2567 | hypothetical protein | 6.57E+06 | 1.55 | 0.00 | S | + |
| TDE2568 | thiamine biosynthesis protein ThiI (thiI) | 0.00E+00 | NA^D^ | 0.00 | H | + |
| TDE2571 | conserved hypothetical protein | 1.04E+07 | 0.00 | 0.01 | S | - |
| TDE2573 | glucose-6-phosphate isomerase (pgi) | 9.75E+06 | 2.08 | 0.03 | G | + |
| TDE2580 | GGDEF domain protein | 3.34E+06 | 0.29 | 0.02 | T | - |
| TDE2591 | rhodanese-like domain protein | 2.63E+07 | 0.62 | 0.01 | P | - |
| TDE2592 | DbpA RNA binding domain protein | 3.12E+07 | 0.58 | 0.01 | S | - |
| TDE2594 | conserved hypothetical protein | 3.91E+06 | 0.00 | 0.00 | S | - |
| TDE2596 | hypothetical protein | 7.30E+06 | 0.19 | 0.01 | S | - |
| TDE2597 | ribulose-phosphate 3-epimerase (rpe) | 1.04E+07 | 0.00 | 0.01 | G | - |
| TDE2602 | outer membrane protein, putative | 2.80E+08 | 0.58 | 0.03 | M | - |
| TDE2611 | conserved hypothetical protein | 2.62E+06 | 0.32 | 0.01 | S | - |
| TDE2614 | ApbE family protein | 1.67E+06 | 0.00 | 0.00 | H | - |
| TDE2616 | cyclic nucleotide-binding protein | 1.17E+07 | 2.23 | 0.02 | T | + |
| TDE2643 | oxidoreductase, FAD-dependent | 0.00E+00 | NA^D^ | 0.00 | C | + |
| TDE2670 | glycosyl transferase, group 4 family protein | 1.35E+06 | 3.20 | 0.01 | M | + |
| TDE2673 | hypothetical protein | 0.00E+00 | NA^D^ | 0.00 | - | + |
| TDE2682 | conserved hypothetical protein | 6.99E+06 | 1.64 | 0.00 | L | + |
| TDE2685 | flagellar synthesis regulator FleN (fleN) | 1.09E+06 | 0.00 | 0.00 | D | - |
| TDE2692 | CTP synthase (pyrG) | 1.61E+07 | 1.54 | 0.01 | F | + |
| TDE2706 | membrane protein, putative | 4.02E+06 | 1.91 | 0.03 | G | + |
| TDE2708 | hypothetical protein | 1.16E+07 | 0.45 | 0.00 | S | - |
| TDE2709 | BNR domain protein | 9.42E+06 | 0.55 | 0.01 | S | - |
| TDE2712 | hypothetical protein | 3.43E+07 | 2.10 | 0.03 | S | + |
| TDE2721 | helicase domain protein | 3.38E+06 | 2.70 | 0.00 | L | + |
| TDE2725 | cyclic nucleotide binding domain/GGDEF domain protein | 3.80E+06 | 0.00 | 0.00 | T | - |
| TDE2730 | hydrolase, TatD family | 4.33E+07 | 0.65 | 0.00 | L | - |
| TDE2734 | hypothetical protein | 3.61E+07 | 0.23 | 0.00 | S | - |
| TDE2748 | acetyltransferase, GNAT family | 3.35E+07 | 0.63 | 0.03 | S | - |
| TDE2761 | conserved hypothetical protein | 1.95E+06 | 0.00 | 0.00 | N | - |
| TDE2762 | flagellar motor switch protein FliY (fliY) | 4.06E+07 | 0.23 | 0.02 | N | - |
| TDE2764 | flagellar protein FliL (fliL) | 6.51E+07 | 0.12 | 0.02 | N | - |
| TDE2765 | flagellar motor rotation protein B (motB) | 3.44E+07 | 0.00 | 0.02 | N | - |
| TDE2766 | motility protein A (motA) | 3.46E+07 | 0.42 | 0.00 | N | - |
| TDE2768 | flagellar hook protein FlgE (flgE) | 4.39E+07 | 0.33 | 0.01 | N | - |
| TDE2771 | conserved hypothetical protein | 0.00E+00 | NA^D^ | 0.04 | - | + |
| TDE2775 | lipoprotein, putative | 8.31E+06 | 0.51 | 0.01 | S | - |
| TDE2776 | proline iminopeptidase (pip) | 2.50E+07 | 1.78 | 0.04 | E | + |
| TDE2779 | hypothetical protein | 5.98E+07 | 0.10 | 0.01 | S | - |
| TDE2783 | methyl-accepting chemotaxis protein | 2.74E+06 | 0.00 | 0.01 | T | - |

^A^ The abundance of each protein in the wild-type *T. denticola* ATCC 33520 was calculated from the average IBAQ intensity from three replicates. Zero value indicates the protein abundance was under the detectable limit.

^B^ Geometric mean of ratios, from three replicates, produced from the LFQ intensity of protein in *∆motB* relative to that of protein in wild-type. Ratio of ≥1.5 indicates that the protein had increased in abundance in *∆motB* relative to wild-type and ratio of ≤0.67 indicates that the protein had decreased in abundance in *∆motB* relative to wild-type. Zero ratio indicates that the protein was identified in ATCC 33520 but not in *∆motB*.

^C^ One-letter abbreviations for the functional COG categories: J, translation, ribosomal structure and biogenesis; K, transcription; L, replication, recombination and repair; D, cell cycle control, cell division, chromosome partitioning; V, defense mechanisms; T, signal transduction mechanisms; M, cell wall/membrane/envelope biogenesis; N, cell motility; U, intracellular trafficking, secretion, and vesicular transport; O, posttranslational modification, protein turnover, chaperones; C, energy production and conversion; G, carbohydrate transport and metabolism; E, amino acid transport and metabolism; F, nucleotide transport and metabolism; H, coenzyme transport and metabolism; I, lipid transport and metabolism; P, inorganic ion transport and metabolism; Q, secondary metabolites biosynthesis, transport and catabolism; R, general function prediction only; S, function unknown.

^D^ NA: Not applicable as the protein was not detected in the wild-type. Refer to Supplementary Table 5 for the protein abundance in *∆motB*.
