# Supplementary material for: The Role of Treponema denticola Motility in Synergistic Biofilm Formation With Porphyromonas gingivalis
Source: Front Cell Infect Microbiol. 2019 Dec 18;9:432. doi: 10.3389/fcimb.2019.00432 (PMC6930189; doi:10.3389/fcimb.2019.00432)
Supplement: Supplementary Table 5 — Proteins that were not detected in T. denticola ATCC 33520 but were detected in ΔmotB (p < 0.05). [file Table_5.DOCX]

**Supplementary Table 5. Proteins that were not detected in *T. denticola* ATCC 33520 but were detected in *∆motB* (*p*<0.05).** Proteins predicted to be organized in an operon were shaded.

| Locus Tag | Protein description | *∆motB* abundance^A^ | COG^B^ |
| --- | --- | --- | --- |
| TDE0385 | ABC transporter, ATP-binding/permease protein | 2.37E+06 | V |
| TDE0607 | ParA family ATPase | 1.26E+06 | D |
| TDE0650 | membrane protein, putative | 2.02E+06 | R |
| TDE0834 | Na^+^-translocating NADH/quinone reductase E subunit (nqrE) | 1.58E+07 | C |
| TDE0983 | oligopeptide/dipeptide ABC permease, frameshift mutation | 1.38E+07 | - |
| TDE0987 | oligopeptide/dipeptide ABC transporter, ATP-binding protein | 1.03E+07 | E |
| TDE1277 | Fe-hydrogenase large subunit family protein | 2.94E+06 | C |
| TDE1388 | conserved hypothetical protein | 5.34E+06 | S |
| TDE1432 | conserved domain protein | 6.79E+05 | S |
| TDE1588 | tryptophanyl-tRNA synthetase, putative | 1.79E+06 | J |
| TDE1713 | hypothetical protein | 4.97E+06 | S |
| TDE1730 | glycosyl hydrolase, family 2 | 1.38E+06 | G |
| TDE2285 | conserved hypothetical protein | 3.74E+06 | S |
| TDE2325 | conserved hypothetical protein | 8.99E+06 | S |
| TDE2568 | thiamine biosynthesis protein ThiI | 1.76E+06 | H |
| TDE2643 | oxidoreductase, FAD-dependent | 1.74E+07 | C |
| TDE2673 | hypothetical protein | 3.83E+07 | - |
| TDE2771 | conserved hypothetical protein | 7.51E+05 | - |

^A^ The abundance of each protein was calculated from the average IBAQ intensity from three replicates.

^B^ One-letter abbreviations for the functional COG categories: J, translation, ribosomal structure and biogenesis; K, transcription; L, replication, recombination and repair; D, cell cycle control, cell division, chromosome partitioning; V, defense mechanisms; T, signal transduction mechanisms; M, cell wall/membrane/envelope biogenesis; N, cell motility; U, intracellular trafficking, secretion, and vesicular transport; O, posttranslational modification, protein turnover, chaperones; C, energy production and conversion; G, carbohydrate transport and metabolism; E, amino acid transport and metabolism; F, nucleotide transport and metabolism; H, coenzyme transport and metabolism; I, lipid transport and metabolism; P, inorganic ion transport and metabolism; Q, secondary metabolites biosynthesis, transport and catabolism; R, general function prediction only; S, function unknown.
