# Supplementary material for: The Role of Treponema denticola Motility in Synergistic Biofilm Formation With Porphyromonas gingivalis
Source: Front Cell Infect Microbiol. 2019 Dec 18;9:432. doi: 10.3389/fcimb.2019.00432 (PMC6930189; doi:10.3389/fcimb.2019.00432)
Supplement: Supplementary file 6 [file Table_2.DOCX]

Name	Minimum	Maximum	Length	Change	Coverage	Polymorphism Type	Strand-Bias	Variant Frequency	Variant P-Value (approximate)	Mutant *T. denticola* 33520 Strain	Amino Acid Change	CDS Position	Codon Change	old locus tag NCBI reference sequence NZ_AGDS00000000.1	new locus tag NCBI reference sequence NZ_KB445542.1	Protein Effect	protein_id	locus tag of gene equivalent in 35405	Protein Identity	Predicted effect on protein structure / function?	Detected by proteomics in wt?	Detected by proteomics in mutant?
A	652,556	652,556	1	G -> A	19	SNP (transition)	78.90%	100.00%	1.00E-57	ΔFlgE	V -> I	4	GTA -> ATA	HMPREF9722_00603	HMPREF9722_RS03030	Substitution	EMB42807.1	TDE2767	flagellar protein-like protein	No effect, conservative change	No	No
G	652,680	652,680	1	C -> G	24	SNP (transversion)	50.00%	100.00%	2.50E-46	ΔFlgE	T -> R	128	ACG -> AGG	HMPREF9722_00603	HMPREF9722_RS03030	Substitution	EMB42807.1	TDE2767	flagellar protein-like protein	No effect, conservative change	No	No
A	652,859	652,859	1	G -> A	32	SNP (transition)	65.60%	100.00%	1.60E-93	ΔFlgE	V -> I	88	GTT -> ATT	HMPREF9722_00604	HMPREF9722_RS03035	Substitution	EMB42808.1	TDE2766	motility protein A motA	No effect, conservative change	Yes	Yes
T	652,888	652,888	1	G -> T	36	SNP (transversion)	61.10%	100.00%	4.00E-69	ΔFlgE	M -> I	117	ATG -> ATT	HMPREF9722_00604	HMPREF9722_RS03035	Substitution	EMB42808.1	TDE2766	motility protein A motA	No effect, conservative change	Yes	Yes
T	652,888	652,888	1	G -> T	38	SNP (transversion)	60.50%	100.00%	6.30E-73	ΔMotB	M -> I	117	ATG -> ATT	HMPREF9722_00604	HMPREF9722_RS03035	Substitution	EMB42808.1	TDE2766	motility protein A motA	No effect, conservative change	Yes	Yes
C	882,559	882,558	0	+C	41	Insertion	51.20%	100.00%	1.30E-119	ΔFlgE		2,710		HMPREF9722_00793	HMPREF9722_RS03985	Frame Shift	EMB42236.1	TDE2566	Hypothetical	Frame shift at amino acid (aa) 904, protein missing last 48 aa, protein truncated at aa 911	Yes	Yes
